# Design of a Chitinase-Responsive, Depolymerizable Petroleum-Derived Polymer for Circular and Antifouling Materials

**DOI:** 10.1021/acsapm.6c00740

**Published:** 2026-06-03

**Authors:** Cody J. Velikaneye, Sarah Kispert, Alexis Pishnyuk, Madison Kajuffa, Devin Fauver, Bisher Lpizra, Sneh Dagar, Safi Dapetel Balkissou Wouna, Kyle P. Buckley, Tarek Ibrahim, Hao Sun, Kristine Horvat, Chong Qiu, Huan Gu

**Affiliations:** Department of Chemistry & Chemical Engineering and Biomedical Engineering, Tagliatela College of Engineering, 8518 University of New Haven, West Haven, Connecticut 06516, United States

**Keywords:** biodegradation, enzyme-responsive, enzyme kinetics, petroleum derived polymer, Chitinase, imide group, smart materials

## Abstract

Petroleum-derived polymers (PDPs), including polyolefins and polyesters, combine mechanical robustness and thermal stability but persist in the environment due to chemically inert C–C backbones. Here, we report PHEVD (poly­[7-(2-hydroxyethyl)-2,4-divinyl-3-oxa-7-azabi­cyclo[3.3.0]­octane-6,8-dione]), a polymer designed to preserve PDP-relevant thermal stability and hydrophobic film performance while incorporating enzyme-addressable imide and amide motifs. Compared with polyethylene terephthalate (PET) and polyethylene (PE), PHEVD exhibits comparable thermal robustness yet undergoes rapid and near-complete depolymerization within 7 days under mild aqueous conditions in the presence of *Pseudomonas aeruginosa* (PAO1), with measurable degradation also observed for *Chlorella vulgaris*. Integrated transcriptomic, mutant, and purified-enzyme analyses implicate Chitinase-associated pathways in degradation. ^1^H NMR and LC-MS confirm the disappearance of parent polymer signals and the formation of low-molecular-weight, soluble products, indicating chemical depolymerization rather than persistent microplastic fragmentation. Identified degradation products correlate with biofilm-dispersion signatures, linking controlled breakdown to functional biological outcomes. By retaining PDP-like performance during use while enabling selective, biologically triggered end-of-life conversion into soluble small molecules, PHEVD demonstrates a structure-guided strategy to reduce environmental persistence relative to conventional PDPs and advance sustainable polymer design.

## Graphical abstract

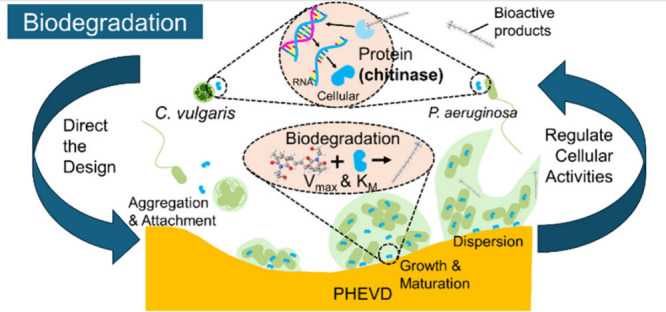

## 1 Introduction

Petroleum-derived polymers (PDPs), including polyethylene (PE), polypropylene (PP), and polyethylene terephthalate (PET), are widely used because they combine durability, versatility, and low cost, but their chemical robustness also contributes to persistent environmental accumulation and a growing plastics burden.^1–3^ Although depolymerization is often proposed as a route toward circularity, native PDPs, particularly polyolefins, remain highly resistant to degradation because their backbones are dominated by strong C–C and C–H bonds.^3–6^ Existing thermal and catalytic approaches often require harsh conditions, while biologically mediated degradation of native PDPs is generally slow and incomplete, proceeding mainly through surface oxidation, fragmentation, or limited erosion rather than selective depolymerization.^3,7,8^

To address this limitation, recent material-design strategies have introduced cleavable motifs into otherwise hydrocarbon-rich polymer structures, including esters, carbonates, acetals, ketals, and other selectively labile linkages.^9–14^ In parallel, enzyme-responsive materials have been successfully developed in non-PDP systems such as hydrogels, nanoparticles, prodrugs, coatings, and textiles, where precise biological triggers can drive controlled structural transformation.^15–21^ Translating this concept to PDP-like materials, however, remains challenging because canonical enzymes have poor access to hydrophobic, crystalline, and chemically inert polyolefin backbones.^14^ As a result, there is increasing interest in designing polyolefin-like polymers that retain useful application-relevant properties while incorporating selectively cleavable motifs that enable triggered degradation under mild conditions.^22^

From a sustainability standpoint, an ideal PDP analogue would combine practical thermal and mechanical stability during use with selective activation, controlled degradation kinetics, and reduced environmental persistence at end of life. Here, we report poly­[7-(2-hydroxyethyl)-2,4-divinyl-3-oxa-7-azabi­cyclo[3.3.0]­octane-6,8-dione] (PHEVD), an enzyme-responsive polymer designed to address this need. We show that PHEVD undergoes degradation under mild biological conditions in the presence of *Pseudomonas aeruginosa* (PAO1) and *Chlorella vulgaris* (*C. vulgaris*). Transcriptomic analysis identifies Chitinase-associated pathways as strongly upregulated during polymer exposure, implicating Chitinase as a key mediator of degradation. Further analyses using purified Chitinase, ^1^H NMR, and LC-MS support progressive chain scission and the formation of low-molecular-weight degradation products rather than simple physical fragmentation. Notably, selected degradation products also disperse mature PAO1 biofilms, linking controlled depolymerization to functional antibiofilm activity. Together, these results establish PHEVD as an enzyme-responsive PDP analogue that couples application-relevant performance with biologically triggered degradation, offering a promising strategy for reduced persistence, controlled end-of-life processing, and sustainable functional materials.

## 2 Results

### 2.1 Structure–Property Relationships Governing PHEVD Surface Hydrophobicity

PHEVD was synthesized via ring-opening metathesis polymerization (ROMP) (Figure S1). Proton nuclear magnetic resonance spectroscopy (^1^H NMR) confirmed the expected polymer structure, showing characteristic olefinic resonances associated with the backbone (Figure S1). Size-exclusion chromatography (SEC) indicated a number-average molecular weight of 81.7 kDa with a narrow dispersity (*Đ* = 1.07), consistent with well-controlled polymerization (Figure S2). Structurally, PHEVD comprises a polyimide-rich backbone containing imide functionalities (−CO–N–CO−) and secondary amide linkages (−CONH−) (Figure 1a,b), which together impart both chemical robustness and enzyme-addressable motifs.

**Figure 1:**
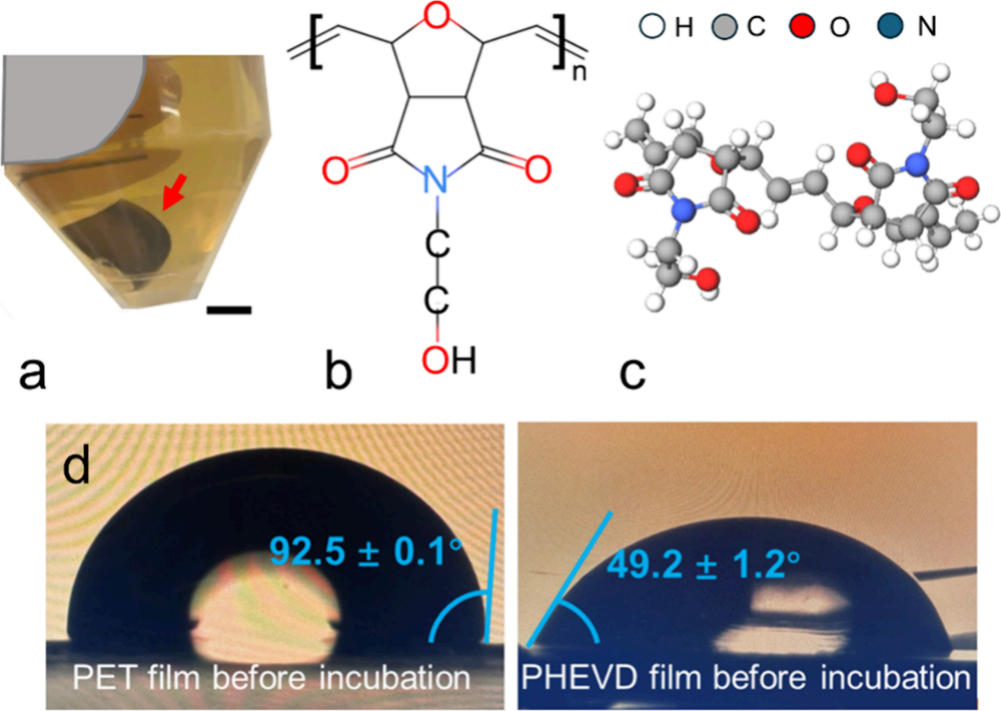
Characterization of PHEVD film. (a) PHEVD film (indicated by red arrow) immersed in 50 mL of sterile Lysogeny Broth (LB) within a falcon tube (scale bar = 4.5 mm). (b) 2D chemical structure of PHEVD, showing a hypothetical Chitinase cleavage site. (c) 3D representation of the PHEVD polymer. (d) Water contact angle on PET and PHEVD films.

Thermal properties were evaluated using differential scanning calorimetry (DSC) and thermogravimetric analysis (TGA). PHEVD exhibited no detectable glass transition temperature and maintained thermal stability up to 375–550 °C, depending on the degradation criterion applied (Table S1). This stability range is comparable to reported thermal degradation onsets for common petroleum-derived polymers, including polyethylene (PE) and polyethylene terephthalate (PET), indicating that incorporation of enzyme-responsive functionalities does not compromise bulk thermal robustness under typical processing or use conditions. Consistent with its chemical composition, the combined presence of imide groups, aliphatic segments, and bicyclic ring carbons renders PHEVD predominantly hydrophobic, analogous to the aromatic-driven hydrophobicity observed in PET. This behavior is supported by a static water contact angle of 92.5 ± 0.1° (>90°). Notably, surface enrichment of amide functionalities significantly alters interfacial properties, reducing the contact angle to 49.2 ± 1.2° (<90°) (Figure 1c,d), demonstrating that PHEVD retains tunable surface chemistry.

### 2.2 Microorganism-Mediated Degradation of PHEVD under Biologically Relevant Conditions

To evaluate whether PHEVD is responsive to microbially secreted enzymes, PHEVD films were first incubated with PAO1 (Figure 2). PAO1 is known to secrete oxidative and hydrolytic enzymes capable of initiating depolymerization of otherwise recalcitrant plastics such as PET, albeit typically with limited mass loss (∼9–20% over 120 days).^3,7^ Polymeric biodegradation under ambient conditions generally proceeds through microbial adhesion and biofilm formation, followed by biodeterioration, biofragmentation, bioassimilation, and mineralization.^8^

**Figure 2:**
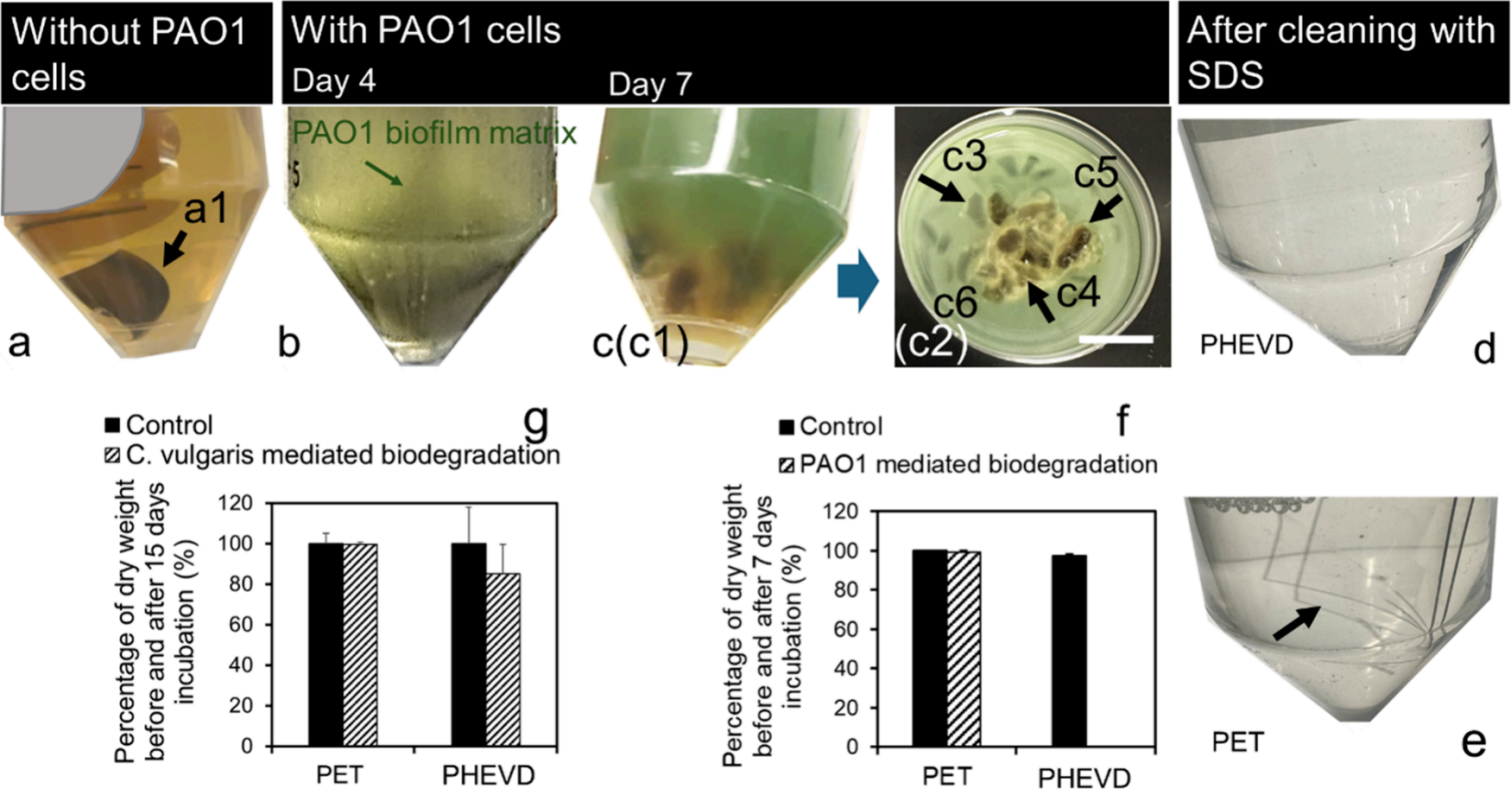
Biodegradation of PHEVD. (a) PHEVD films ((a1), brown) incubated in LB without microbes for 7 days as control. (b) PHEVD films after 4 days of incubation with PAO1 cells at 37 °C with shaking at 200 rpm. The PHEVD film is fully wrapped in PAO1 biofilms. (c) PHEVD films after 7 days of incubation with PAO1 cells. (c2) The solution in (c1) is poured into a 35 mm Petri dish, showing that the PHEVD film in (a) is decomposed into transparent (T) debris (c3), medium-transparent (MT) debris (c4), nontransparent (NT) debris (c5), and bacterial solutions (c6). (d, e) PHEVD residues (shown in (c, d)) and PET residues (e) in 2% (w/v) SDS solution after three washes with sterile DI water and 4 h of incubation in SDS solution at 37 °C with shaking at 200 rpm. The arrow in (e) highlights the remaining PET films. (f) Change of the dry weight of PET and PHEVD films after 7 days of incubation in LB with and without PAO1 cells. (g) Change of the dry weight of PET and PHEVD films after 15 days of incubation in BBM with and without *C. vulgaris* (*N* ≥ 3).

Upon exposure to PAO1 (Figure 2a), PHEVD supported substantially greater biofilm accumulation than PET after 2 days at 37 °C with shaking (200 rpm) (Figure 2b). On PHEVD surfaces, biofilm development was accompanied by visible film swelling by day 4 and progressive disruption of the three-dimensional (3D) film structure by day 7 (Figure 2b,c). Notably, as degradation progressed, metabolic byproducts coincided with the complete detachment of cells from the PHEVD surface by day 6, consistent with the loss of structural integrity. After 7 days in PAO1 solutions, three washes with deionized (DI) water, and 4 h of incubation in 2% (w/v) sodium dodecyl sulfate (SDS) solution at 37 °C with shaking at 200 rpm, PHEVD exhibited 100% dry-weight reduction (Figure 2d), whereas the PET (PDP control) and high-density PE (HDPE, polyolefin control) showed only a marginal decrease of 0.6 ± 0.3% and 0.5 ± 2.8% (Figure 2e,f and Figure S3), respectively. Under these conditions, the degradation rate of PHEVD in LB medium was 0.74 ± 0.20 mg h^–1^, approximately 40-fold higher than that observed for PET (0.02 ± 0.01 mg h^–1^) and HDPE (0.02 ± 0.00 mg h^–1^). To investigate if microplastics were formed after PAO1-mediated degradation, we measured the turbidity of SDS solutions with PHEVD residues at an optical density of 510 nm (OD_510_) and imaged the SDS solutions with a 40× lens and Echo fluorescence microscope. By comparing the turbidity and bright-field images of the SDS solutions with PHEVD residues with the results of clean SDS solutions, no significant changes were observed in turbidity (OD_510_ of 0.158 ± 0.006 and 0.166 ± 0.022, *p* = 0.57 > 0.05) and bright-field images (Figure S4), suggesting that PHEVD films were converted into products that are soluble in SDS solutions instead of microplastics.

To assess whether PHEVD susceptibility extended beyond bacterial systems, PHEVD films were also incubated with the microalga *C. vulgaris* in Bold’s Basal Medium (BBM) (Figure 2g). After 7 days, *C. vulgaris* induced 15.0 ± 14.6% mass loss of PHEVD, compared to 0.36 ± 1.12% for PET under identical conditions. Although less pronounced than degradation mediated by PAO1, these results are consistent with a preliminary trend observed with PAO1 cells, while remaining substantially more resistant to breakdown in the absence of specific biological activity.

### 2.3 Chemical Evidence for PHEVD Depolymerization via ^1^H NMR

To distinguish chemical depolymerization from physical fragmentation, ^1^H NMR spectroscopy was used to analyze PHEVD prior to microbial exposure (Figure 2a1) and the materials recovered after biodegradation (Figure 2c3–c6). Following 7 days of incubation, four distinct fractions were obtained based on visual appearance and biofilm association: transparent (T) products lacking PAO1 biofilms (Figure 2c3), medium-transparent (MT) products containing PAO1 biofilms (Figure 2c4), nontransparent (NT) products (Figure 2c5), and the culture supernatant from PAO1 incubations (Figure 2c6).

As shown in Figure 3, comparison of the ^1^H NMR spectra revealed that the characteristic resonances associated with the intact PHEVD backbone (Figure 2a1) were no longer detectable in the transparent fraction (Figure 2c3), indicating near-complete chemical conversion of the polymer under these conditions. In contrast, partial conversion was observed for the MT and NT fractions (Figure 2c4,c5), where residual PHEVD signals remained, suggesting incomplete degradation and the coexistence of polymeric and lower-molecular-weight species. These results are consistent with spatially heterogeneous biodegradation being influenced by biofilm coverage and local enzymatic activity. These chemical transformations are accompanied by pronounced microscale structural changes in the polymer matrix, as visualized by fluorescence microscopy, shown below.

**Figure 3:**
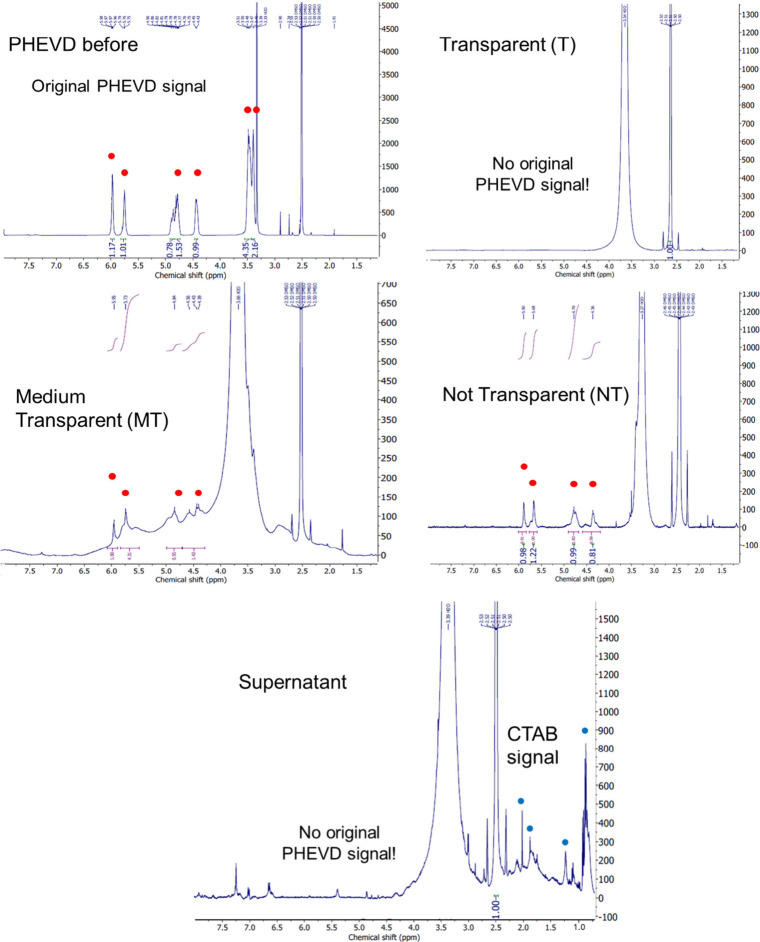
^1^H NMR spectroscopy analysis of PHEVD and its biodegradation products.

All biodegradation-derived samples (Figure 2c4–c6) were readily soluble in 2% (w/v) SDS solution, whereas pristine PHEVD (Figure 2a1) was not, further supporting substantial chemical modification of the polymer during microbial treatment. Notably, ^1^H NMR analysis of the PAO1 culture supernatant (Figure 2c6) suggested the presence of cetyl­tri­methyl­ammon­ium bromide (CTAB), a cationic surfactant known for antimicrobial activity.^30^ The emergence of such bioactive small-molecule species provides a plausible explanation for the observed antibiofilm and antifouling effects of PHEVD degradation products, linking polymer depolymerization to functional biological outcomes.

### 2.4 Biofilm-Associated Disruption of PHEVD Film Structure during PAO1-Mediated Degradation

To examine PAO1-associated degradation of PHEVD at the microscale, PAO1 biofilm cells were fluorescently labeled with acridine orange (AO) and imaged using 3D fluorescence microscopy (Echo Revolve) with GFP filter sets (excitation: 457–487 nm; emission: 502–538 nm) (Figure 4). Two-dimensional (2D) and reconstructed 3D images revealed that, relative to PHEVD films not exposed to PAO1, biofilm-covered PHEVD films underwent pronounced structural disruption following 7 days of incubation. Specifically, intact films were transformed into porous, swollen structures consistent with the hydrogel-like morphology (Figure 4b).

**Figure 4:**
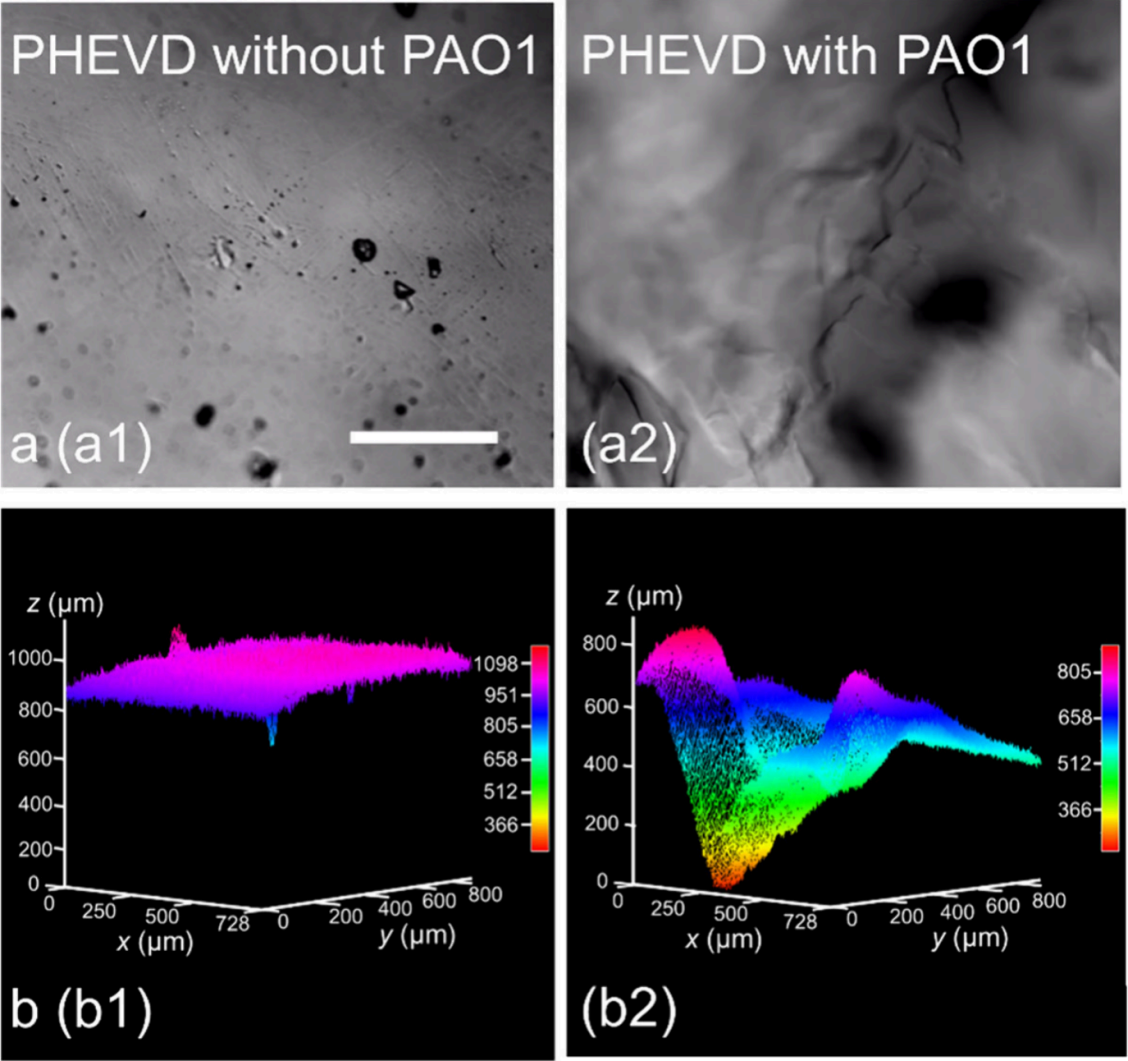
PHEVD surfaces after 7 days of incubation in LB with and without PAO1 cells. (a) Two-dimensional (2D) images of PHEVD surfaces after 7 days of incubation in LB without (a1) and with (a2) PAO1 cells, prior to washing with 2% (w/v) SDS solution. Each 2D image is a merged image obtained using two illumination modes: bright-field for PHEVD and GFP fluorescence for PAO1 cells. (b) Three-dimensional (3D) images corresponding to the surfaces shown in panel (a). Colors indicate surface depth.

These PAO1-exposed PHEVD hydrogels were fully soluble in 2% (w/v) SDS, whereas unexposed PHEVD films remained insoluble under identical conditions. To ensure that observed mass loss reflected polymer degradation rather than residual biomass, both PET and PHEVD films were treated with 2% (w/v) SDS at 37 °C for 4 h with shaking at 200 rpm to lyse and remove bacterial cells prior to dry-weight measurements. SDS treatment had no measurable effect on PET films or on PHEVD films that had not undergone 7 days of incubation, indicating that the postincubation solubility and mass loss of PHEVD arise from PAO1-associated chemical and structural modification rather than surfactant-induced dissolution alone.

Notably, after 7 days of incubation, no intact PAO1 biofilm cells were detected on the surface of the degraded PHEVD hydrogels, as evidenced by the absence of a green fluorescence signal in merged bright-field and fluorescence images (Figure 4b). This observation suggests that degradation-associated changes in the polymer matrix and/or the accumulation of degradation products promote biofilm detachment, further linking the biofilm-mediated structural disruption of PHEVD to its observed antibiofilm behavior.

### 2.5 Transcriptomic Analysis Identifies Enzyme-Associated Pathways Linked to Hydrolytic and Oxidative Degradation

To identify biological pathways associated with the observed depolymerization behavior, rather than assign single-enzyme causality, RNA sequencing (RNA-seq) was performed to identify differentially expressed genes and biological pathways associated with PAO1-mediated degradation after 4 and 6 days of incubation (Figure 5) because PHEVD exhibited stronger and more rapid degradation in the presence of *Pseudomonas aeruginosa* PAO1 than *C. vulgaris*. Transcriptomic analysis revealed multiple pathways that were significantly altered during polymer exposure (log_2_ fold change > 1.0, adjusted *p* < 0.05) (Tables S2–S5), implicating both hydrolytic and oxidative processes in PHEVD degradation.

**Figure 5:**
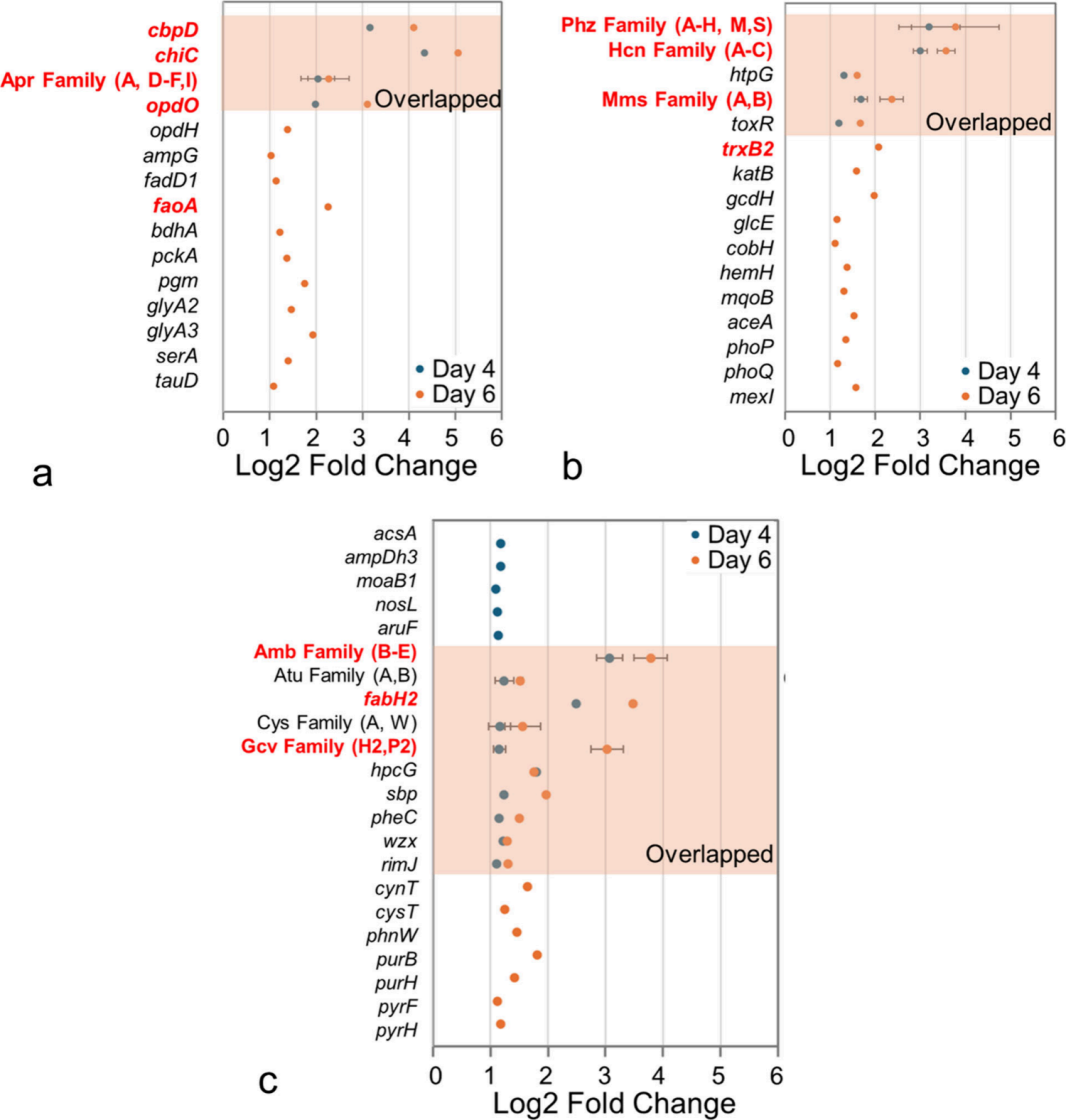
Genes/families in PAO1 that are upregulated during the biodegradation of PHEVD after 4 and 6 days of incubation. (a) Changes of genes/families that are involved in the hydrolytic degradation of amide bonds. (b) Changes of genes/families that are involved in oxidative degradation of the imide ring. (c) Changes of genes/families that are involved in the metabolic assimilation of degradation products (*N* = 3).

Genes associated with hydrolytic processing of amide-containing substrates were prominently upregulated (Figure 5a and Table S2). Notably, *cbpD*, *chiC*, *opdO*, and members of the *apr* gene family exhibited log_2_ fold changes greater than 2.0. After 4 days of incubation, *cbpD* (log_2_FC = 3.17, adjusted *p* = 4.34 × 10^–11^), *chiC* (log_2_FC = 4.33, adjusted *p* = 1.12 × 10^–15^), and *apr* family genes (log_2_FC = 2.04) were strongly upregulated. These genes encode chitin-binding proteins, chitinases, and alkaline proteases, respectively, enzyme classes known to hydrolyze amide-containing substrates and potentially target chemically labile amide linkages within the PHEVD side chains.^31–33^ Expression of these genes increased further by day 6, indicating sustained involvement throughout the degradation process. In addition, several genes not directly associated with amide hydrolysis were upregulated at later time points, suggesting indirect roles in the processing or transport of degradation intermediates.

Genes implicated in oxidative metabolism were also significantly enriched during PHEVD exposure (Figure 5b and Table S3). Members of the *phz*, *hcn*, and *mms* gene families (log_2_FC > 2.0) were upregulated after 4 days, including *phz* (log_2_FC = 2.87 ± 0.62), *hcn* (log_2_FC = 2.99 ± 0.15), and *mms* (log_2_FC = 1.69 ± 0.14). These pathways are involved in phenazine and hydrogen cyanide biosynthesis, both of which are associated with reactive oxygen species (ROS) generation. ROS-mediated oxidative stress may contribute to destabilization of the imide ring structure in PHEVD, facilitating subsequent chain scission.^34,35^ Expression of these oxidative pathways increased further by day 6, consistent with a progressive oxidative contribution to polymer degradation. Additional genes upregulated at later time points may support detoxification, redox balancing, or metabolism of small oxidative degradation products.

Finally, genes involved in the assimilation and metabolic utilization of low-molecular-weight degradation products were significantly upregulated (Figure 5c and Table S4). These included *fabH2* and members of the *amb* and *gcv* gene families (log_2_FC > 2.0). After 4 days, *fabH2* (log_2_FC = 2.50, adjusted *p* = 2.17 × 10^–11^), *amb* (log_2_FC = 2.69 ± 0.87), and *gcv* (log_2_FC = 1.16 ± 0.11) showed elevated expression, suggesting the assimilation of aminobutyric acid, acetate, or related small molecules derived from PHEVD side chains or backbone fragments. These metabolic pathways were further upregulated at day 6, supporting their role in sustaining microbial growth during polymer degradation.^36–38^

### 2.6 Evidence for Chitinase-Associated Responsiveness of PHEVD

Transcriptomic analysis identified *chiC*, encoding Chitinase, as one of the most strongly upregulated genes during PAO1-mediated degradation of PHEVD. This observation suggests that Chitinase-associated pathways play a significant role in the biological response to PHEVD exposure. Upregulation of *chiC* was independently validated by RT-qPCR at both day 4 and day 7 (log_2_FC = 2.58 ± 1.68 and 3.93 ± 5.48, respectively), confirming sustained transcriptional activation. Chitinases are widely secreted by microorganisms in natural environments to hydrolyze chitin and other amide-containing biopolymers, making them plausible contributors to PHEVD degradation.^31,32,39^

To directly assess the functional relevance of *chiC*, biodegradation experiments were conducted using a PAO1 Δ*chiC* knockout strain. Deletion of *chiC* resulted in a significant reduction in the PHEVD degradation rate, from 0.74 ± 0.20 mg h^–1^ for the wild-type strain to 0.28 ± 0.03 mg h^–1^ for the mutant (*p* = 0.0008) (Figure 6a). While degradation was not fully abolished, this decrease demonstrates that Chitinase activity contributes substantially to PHEVD depolymerization, while also indicating that additional enzymes or pathways may participate in the overall process.

**Figure 6:**
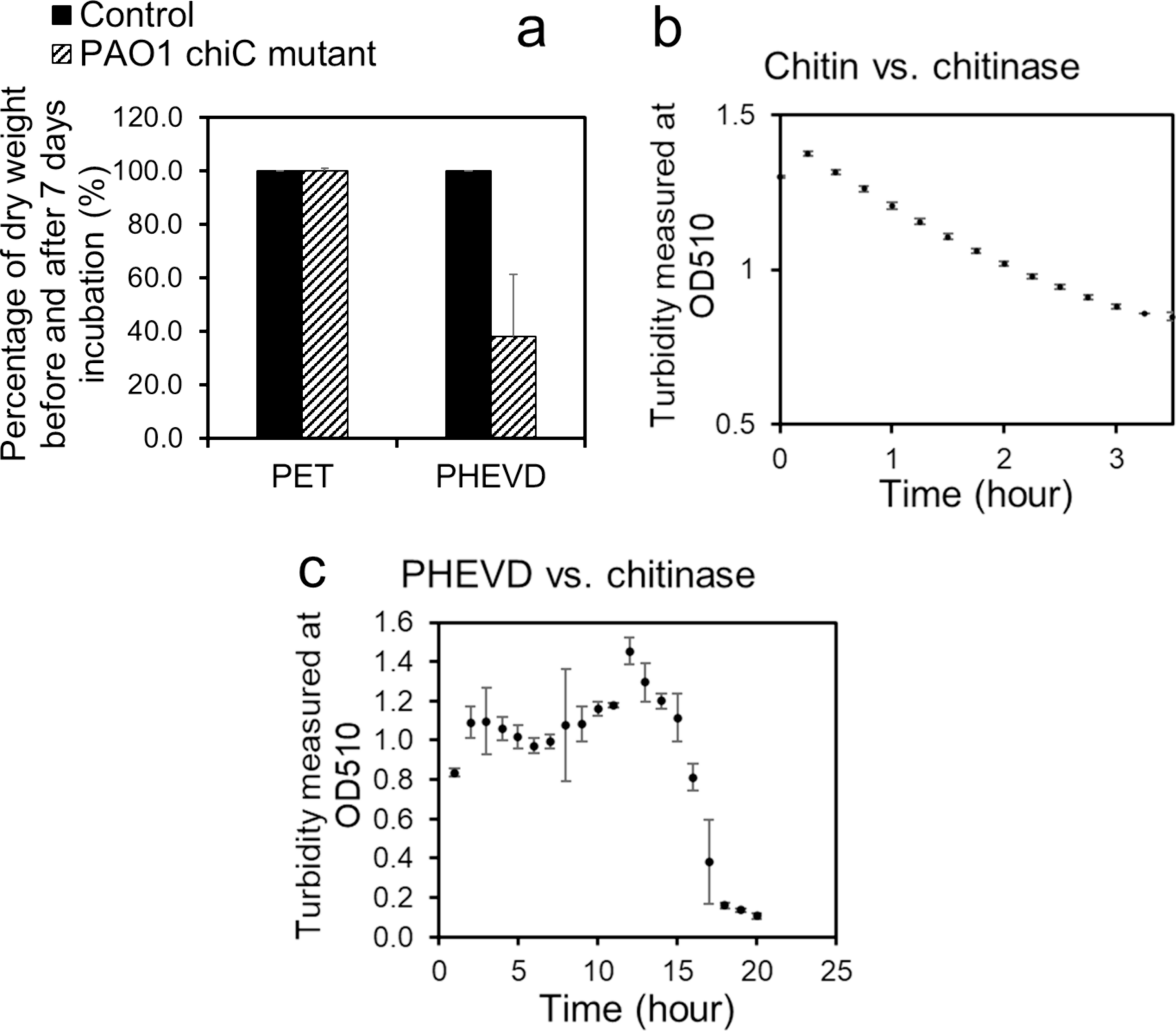
Chitinase response of PHEVD. (a) Change of the dry weight of PET and PHEVD films after 7 days of incubation in LB with and without PAO1 cells. (b, c) The change of turbidity of chitin and Chitinase (b) and PHEVD and Chitinase (c) mixtures over time at 37 °C measured at an optical density of 510 nm (*N* ≥ 3).

To further probe enzyme–polymer interactions in isolation from cellular processes, PHEVD particles were incubated with purified Chitinase at 37 °C, using colloidal chitin as a positive control (Figure 6b,c). Changes in suspension turbidity, measured at an optical density of 510 nm, were analyzed using a Michaelis–Menten kinetic framework, where substrate concentration ([*S*]) and degradation rate (−*r*_S) were related according to established models.^40^ Since this assay does not directly measure chemical bond-cleavage kinetics and may also reflect changes in particle dispersion, aggregation, scattering behavior, or effective surface area during incubation, the fitted parameters are presented only as empirical descriptors of apparent suspension response rather than as rigorous catalytic constants for polymer depolymerization. Under these conditions, fitting the experimental data yielded apparent parameters for PHEVD particles of *V*_max_ = 359.3 μg h^–1^ and *K*_m_ = 37.8 mg mL^–1^, compared to *V*_max_ = 5.35 μg h^–1^ and *K*_m_ = 1.53 mg mL^–1^ for colloidal chitin. These differences in parameters between PHEVD and chitin suggest that Chitinase exposure is associated with measurable time-dependent physical changes in PHEVD particle suspensions, with the higher *K*_m_ for PHEVD being consistent with less favorable initial enzyme–substrate interaction or accessibility than for native chitin.

For enzyme assays, both PHEVD and PET films were converted into particles with average diameters between 0 and 10 μm (Figure S5) to enable comparison with powdered colloidal chitin. Notably, polymer morphology and incubation history significantly influenced enzymatic responsiveness. While PET films were resistant to both PAO1-mediated degradation (Figure 2e) and purified Chitinase (Figure S6), PET particles exhibited gradual loss upon prolonged incubation with Chitinase at 37 °C (Figure S7), indicating that the OD_510_ assay is sensitive to particle-level physical changes that are not uniquely attributed to substrate-specific bond cleavage. The turbidity loss, in conjugation with the reduced degradation observed in the *ΔchiC* mutant, the transcriptomic upregulation of Chitinase-associated pathways, the altered solubility of degradation products, and the ^1^H NMR and LC-MS results, supports Chitinase as a major contribution to PHEVD degradation within a broader multienzyme degradation network.

### 2.7 Antibiofilm Activity Associated with PHEVD Biodegradation Products

Since the detachment of PAO1 biofilms cells was observed on day 6 of biodegradation, we examined the antibiofilm activity of the PHEVD biodegradation products (Figure 2c,c2). Transcriptomic analysis further revealed that genes associated with biofilm regulation and cellular stress responses were differentially expressed during PAO1-mediated degradation of PHEVD. After 6 days of incubation, genes implicated in biofilm dispersion, including *fliC* (log_2_FC = 1.20 ± 0.002) and *bdlA* (log_2_FC = 1.52 ± 1.40 × 10^–7^), were significantly upregulated (Figure 7a and Table S5). In parallel, multiple detoxification- and stress-response pathways were enriched (Figure 7b), suggesting that degradation-associated products influence biofilm stability and cellular homeostasis. Together, these transcriptional changes are consistent with enhanced biofilm dispersal rather than sustained biofilm maturation in the presence of PHEVD degradation products.

**Figure 7:**
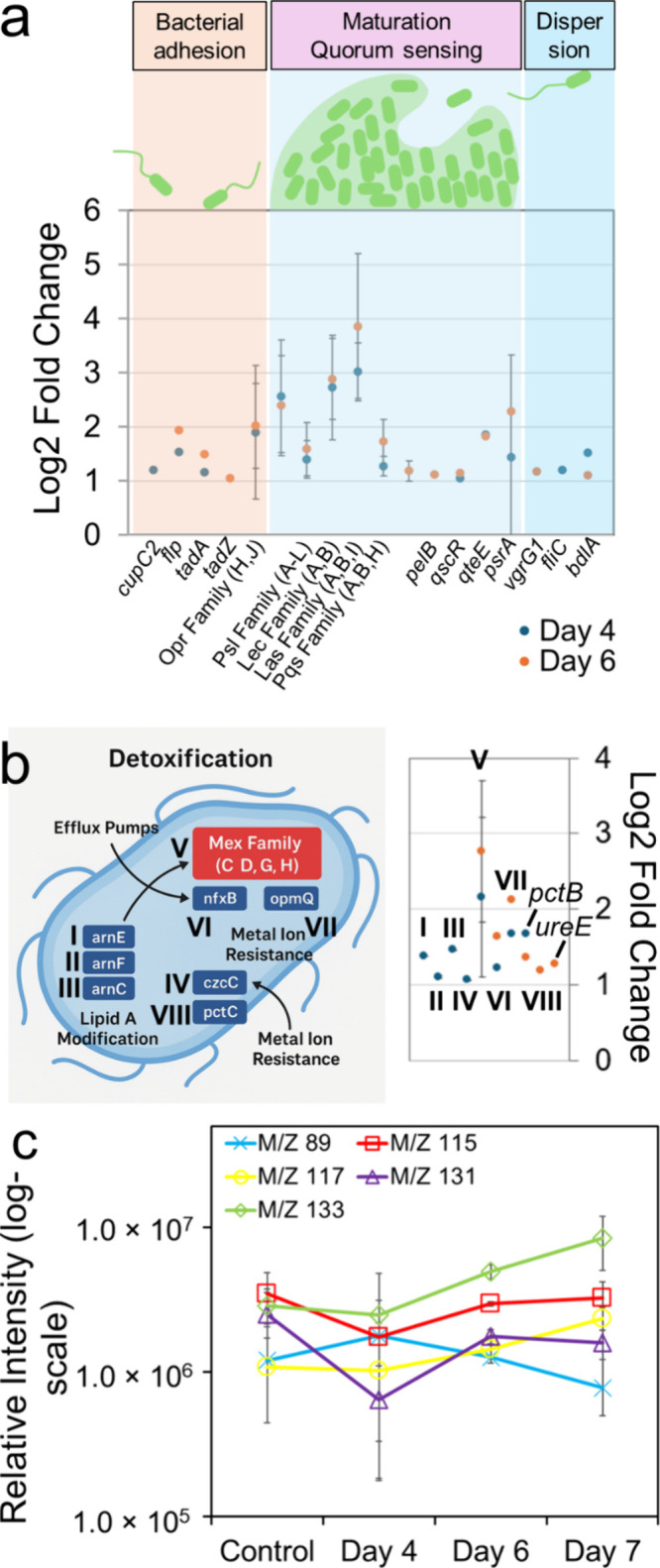
PHEVD biodegradation products showed biofilm dispersion and stress response. (a) Biofilm-associated genes/families that are upregulated in PAO1 cells with PHEVD. (b) Genes/families that are involved in detoxification in PAO1 cells during degradation. (c) Validation of bioactive products using LC-MS (*N* = 3).

To chemically characterize these products, ^1^H NMR spectroscopy (Bruker 400 MHz) revealed resonances in the δ = 7.60–7.67 ppm range that were absent in pristine PHEVD and increased progressively over the 7 day incubation period (Figure 7c). Complementary LC-MS analysis (Agilent 6120B) detected low-molecular-weight species with *m*/*z* values of 115, 117, and 131, suggesting the formation of small organic acids, including maleic acid,^41^ succinic acid,^42^ and potentially glutaric acid.^43^ These compounds are expected products of hydrolytic and enzyme-mediated cleavage of maleimide-containing polymer segments and are known to influence microbial physiology and biofilm behavior. While these metabolites are not presented as broad-spectrum antimicrobials, their accumulation provides a plausible chemical basis for the observed transcriptional signatures associated with biofilm dispersion and stress response.

## 3 Discussion

Enzyme-responsive materials that directly interface with living systems offer a distinct advantage over conventional stimuli-responsive platforms, as they exploit endogenous biological activity to trigger material transformation without external inputs.^44–46^ This feature is particularly compelling for petroleum-derived polymers, whose durability underpins widespread use but also drives environmental persistence.^47,48^ Despite extensive work on enzyme-responsive hydrogels, coatings, and biomedical polymers,^44,49–51^ PDP-like materials that combine industrially relevant performance with selective, biologically triggered depolymerization remain rare. The present study addresses this gap by demonstrating how the targeted incorporation of enzyme-addressable motifs into a PDP can decouple in-use stability from end-of-life degradability.

PHEVD was designed to retain key performance attributes of conventional PDPs while enabling enzyme-mediated breakdown. The imide-rich backbone introduces rigid, planar segments that function analogously to aromatic units in PET, conferring hydrophobicity, thermal robustness, and dimensional stability.^52,53^ Differential scanning calorimetry (DSC) and thermogravimetric analysis (TGA) showed that PHEVD exhibited no detectable glass transition and maintains thermal stability up to 375–550 °C, comparable to reported degradation onsets for PE^54,55^ and PET.^56,57^ These data indicate that the incorporation of secondary amide and imide functionalities does not compromise bulk thermal performance under typical processing or service conditions. At the interface, PHEVD displays tunable wettability: while the bulk material is hydrophobic, surface enrichment of amide functionalities yields a hydrophilic state, a combination that distinguishes PHEVD from many degradable polymer alternatives that sacrifice performance for degradability.^58^

These interfacial and structural features directly influence biological interactions. PAO1 rapidly colonized PHEVD surfaces, forming dense biofilms that coincided with film swelling, loss of three-dimensional integrity, and complete mass loss within 7 days, behavior not observed for PET under identical conditions. Preferential PAO1 adhesion likely reflects favorable interactions between hydrophilic PHEVD domains and sugar-rich lipopolysaccharides,^59^ as well as potential chemoattraction to amide-associated nitrogen sources.^60^ In contrast, the more hydrophobic microalga *C. vulgaris* exhibited weaker adhesion and slower degradation, consistent with reports that algal attachment favors hydrophobic substrates,^61,62^ yet still degraded PHEVD more effectively than PET, underscoring the broad biological accessibility of the polymer.

Transcriptomic analysis indicates that PHEVD degradation arises from coordinated hydrolytic, oxidative, and metabolic responses rather than a single-enzyme mechanism. Among these, Chitinase-associated genes, particularly *chiC*, were consistently and strongly upregulated, a finding supported by mutant and purified-enzyme assays. Chitinases are widely distributed environmental enzymes best known for cleaving β-linked amide-containing polysaccharides but increasingly recognized for broader substrate promiscuity.^63,64^ Together, these results establish PHEVD as a rare example of a Chitinase-responsive PDP, expanding the known substrate scope of Chitinase and identifying imide–amide architectures as programmable enzyme-recognition motifs.

A central concern in degradable plastics is whether materials fragment into persistent microplastics rather than undergoing chemical breakdown.^65–67^ Multiple lines of evidence indicate that PHEVD degradation proceeds beyond physical fragmentation. Proton NMR showed the complete disappearance of parent polymer signals in fully degraded samples (Figure 2c3), while LC-MS identified low-molecular-weight products including maleic and succinic acids. Degraded PHEVD films transformed into water-soluble hydrogels and were fully dissolvable in SDS, in contrast to intact PHEVD or PET controls. These observations support a degradation pathway dominated by chain scission and solubilization rather than the accumulation of microplastic debris. Moreover, upregulation of metabolic assimilation pathways suggests that at least a fraction of degradation products enter microbial metabolism, further reducing environmental persistence. Direct time-resolved SEC/GPC, NMR, or LC-MS analysis of purified fractions would be valuable future work for more definitively resolving molecular-weight evolution and bond-cleavage kinetics during Chitinase-mediated degradation.

Beyond depolymerization, PHEVD degradation yields metabolites that have functional ecological consequences. Late-stage upregulation of biofilm dispersion and detoxification genes coincided with the detachment of PAO1 biofilms from degrading PHEVD surfaces. Identified products provide a mechanistic basis for this behavior: maleic acid can disrupt enzymatic activity and redox balance,^68,69^ while succinic acid suppresses biofilm formation through acidification and metabolic interference.^70^ Their gradual accumulation suggests a sustained-release profile that may limit recolonization without acute toxicity.

From a sustainability perspective, PHEVD offers several improvements over traditional PDPs: retention of PDP-like thermal and interfacial performance during use; enzyme-triggered depolymerization under mild, environmentally relevant conditions; conversion into small, soluble molecules rather than persistent microplastics; and generation of degradation products that actively suppress biofilm formation. Although a full life cycle assessment remains beyond the scope of this study, these attributes collectively align with emerging design principles for sustainable plastics and circular polymer systems.

Together, these findings establish PHEVD as a structure-guided, enzyme-responsive PDP prototype and suggest a generalizable design strategy in which selective biological triggers govern the end-of-life behavior. By coupling PDP with programmable enzymatic responsiveness and functional degradation products, this work outlines a path toward sustainable plastics aligned with both materials engineering and biological processes.

## 4 Conclusion

We report PHEVD as an enzyme-responsive petroleum-derived polymer that decouples in-use performance from end-of-life degradability. Incorporation of imide and secondary amide motifs preserves thermal robustness and hydrophobicity comparable to those of conventional PDPs (e.g., PE and PET) while enabling tunable interfacial chemistry. In contrast to PET and HDPE, PHEVD undergoes rapid depolymerization under mild, aqueous conditions in the presence of PAO1. Integrated transcriptomic, mutant, enzymatic, and molecular analyses indicate that degradation proceeds through coordinated hydrolytic and oxidative pathways with Chitinase playing a significant role. Crucially, depolymerization is characterized by the disappearance of parent polymer signals and the formation of low-molecular-weight, soluble products rather than persistent particulate fragments, addressing concerns associated with microplastic accumulation. Identified metabolites correlate with biofilm-dispersion signatures, suggesting functional biological consequences of degradation. Collectively, PHEVD demonstrates the retention of PDP-like performance during use, selective enzyme-triggered breakdown under environmentally relevant conditions, and conversion into soluble small molecules instead of long-lived debris. These findings establish a structure-guided strategy for designing depolymerizable PDPs that integrate material performance with biologically programmable end-of-life pathways.

## 5 Materials and Methods

### 5.1 Synthesis of Monomer HEVD (7-(2-Hydroxyethyl)-2,4-divinyl-3-oxa-7-azabi­cyclo[3.3.0]­octane-6,8-dione)

To a dry flask, exo-7-oxabi­cyclo[2.2.1]­hept-5-ene-2,3-dicarboxylic anhydride (4.00 g, 1.0 equiv, 24.1 mmol) was suspended in anhydrous methanol (100 mL). Then triethylamine (3.68 mL, 1.1 equiv, 26.5 mmol) and 2-aminoethanol (1.45 mL, 1.0 equiv, 24.1 mmol) were sequentially added to the reaction dropwise. The solution was stirred and refluxed for 12 h at 75 °C. The solution was then allowed to cool to room temperature, concentrated under reduced pressure, and then stored at −10 °C for 8 h. The product crystallized from solution, forming large white crystals. The product was filtered, washed with ice-cold methanol, and dried (3.23 g, 64%). The synthesis of HEVD was confirmed by ^1^H NMR (Figure S8).

### 5.2 Synthesis of PHEVD

In a typical procedure for the synthesis of P1 (targeted DP = 100), Grubbs catalyst third generation (12.6 mg, 0.0144 mmol, 1.0 equiv) and anhydrous DMF (1 mL) were added into a 10 mL vial. In a separate vial, HEVD (300 mg, 1.44 mmol, 100 equiv) was fully dissolved in anhydrous DMF (2 mL). Following that, the monomer solution was quickly added into the catalyst solution during stirring. The polymerization was further left to stir for 4 h at room temperature. Finally, ethyl vinyl ether (140 μL, 1.44 mmol, 100 equiv) was added to terminate the reaction. The polymer product was recovered by precipitation into cold methanol.

### 5.3 Thermal Characterizations of PHEVD

A Q-600 model instrument (TA Instruments) was used to perform simultaneous TGA and DSC measurements on the PHEVD materials from room temperature to 600 °C. A clean, empty aluminum sample pan (40 μL) was first placed on the sample holder to tare the balance of its mass at room temperature. A 5–10 mg PHEVD sample, cut into small strips, was added to the pan and then analyzed using the thermal sequence of thermal equilibration at room temperature for 3 min followed by a temperature ramp to 600 °C at 10 °C/min. A significant mass loss of 80 ± 1 wt % at 550 °C was observed; therefore, hermetically sealed sample pans were not used. The instrument performance was evaluated using a calcium oxalate monohydrate standard (with a purity of 99.99%) in the same experimental temperature range. The systematic uncertainties of the TGA and DSC measurements were below 1.1% and 5.7%, respectively.

### 5.4 Microorganisms and Culture

PAO1 and *ΔchiC* mutant were purchased from the Salipante Laboratory in the Department of Laboratory Medicine and Pathology at the University of Washington (Seattle, WA, USA).^71^ PAO1 and its mutant were inoculated from single colonies on less than 14 day old Lysogeny Broth (LB) Agar plates (10 g/L NaCl, 5 g/L yeast extract, 10 g/L tryptone, and 15 g/L agar) and cultured in LB at 37 °C with shaking at 200 rpm. The *C. vulgaris* was purchased from Carolina Biological Supply (15-2068, Burlington, NC, USA). They were cultured in Bold’s Basal Medium (BBM) at 22 °C under a high light level of 200 to 400 foot-candles of fluorescent light 18 to 24″ from the culture. The overnight cultures of PAO1 and its mutant were used to inoculate biofilm growth.

### 5.5 Biodegradation Assay

#### 5.5.1 PHEVD, PET, and HDPE Film Sterilization

PHEVD was pressed into films after synthesis. We prepared PET specimens (Aquafina bottle body), HDPE specimens (Aquafina bottle cap), and PHEVD films for biodegradation assays by cutting films into large pieces. The dry weight of each piece was weighed to 0.001 g using a calibrated, leveled analytical balance before processing. Each specimen was placed in a labeled 50 mL polypropylene conical tube (Fisher Scientific, Hampton, NH, USA) for processing.

For surface sterilization, we added 70% isopropanol (IPA, Sigma-Aldrich, St. Louis, MO, USA) directly to each tube, capped and inverted each tube 10 times to wet the specimen and tube interior fully, and soaked them for 30 min at room temperature. IPA was then carefully decanted to solvent waste by loosening the cap without contacting the specimen or interior. Conical tubes were placed upright with caps loosely threaded and dried overnight (12–16 h) at 60 °C, cooled to room temperature, and reweighed using the same tare method to obtain the postwash, postdry mass. Throughout, we used sterile DI water, kept tubes capped except during addition/decanting to avoid contamination, and verified that specimens remained as single large pieces.

#### 5.5.2 Biofilm Inoculation and Growth

Sterile 50 mL conical tubes containing the precleaned PET body, HDPE cap, and PHEVD film specimens were later used for biofilm inoculation and growth. For PAO1, an overnight culture of PAO1 (prepared as above) was used for biofilm inoculation. We dispensed 15 mL of fresh LB into each tube containing specimens. The optical density of PAO1 cultures at a wavelength of 600 nm (OD_600_) was measured using a spectrophotometer. We inoculated the fresh LB in each tube to a starting OD_600_ of 0.05 with the PAO1 overnight culture. The opening of each tube was covered with a sterile filter and sealed with Parafilm to prevent gaps. Conical tubes were then placed in the 37 °C shaker with a slight angle and shaken at 200 rpm for 7 days. The spent PAO1 cultures in conical tubes were replaced with 15 mL of fresh LB every 2 days. For *C. vulgaris*, 15 mL of *C. vulgaris* cultures in BBM was directly placed in the sterile conical tubes with specimens for 15 days at 22 °C under a high light level of 200 to 400 foot-candles of fluorescent light 18 to 24″ from the culture.

#### 5.5.3 Dry-Weight Quantification

After 7 days of incubation, we removed the inoculated 50 mL conical tubes from the shaker/incubator, took off the Parafilm and filters, and recapped the tubes. We loosened the caps and decanted spent cultures into a designated waste container, ensuring that the specimens remained in the conical tubes. We rinsed each tube three times using DI water. After rinsing, some PHEVD residues were taken for fluorescence imaging to characterize the amount of remaining PAO1 mature biofilms after a 7 day incubation with PAO1 cells. For the PHEVD residues that were used for dry-weight quantification, we added 2% (w/v) SDS solutions to each tube, recapped the tubes tightly, and returned them to the shaker/incubator for 4 h at 37 °C with shaking (200 rpm). After 4 h of incubation, we removed the tubes, discarded SDS into the waste container, and repeated the DI water triple-rinse as mentioned above. After dumping the last rinse DI water, we opened the caps and placed the tubes (with specimens) in a 60 °C oven to dry overnight. The next day, we removed the specimens from the tubes and weighed each on an analytical balance (only the specimens were weighed, not the tube). Finally, we compared postbiodegradation masses to the prebiodegradation masses recorded in section 5.5.1.

### 5.6 Fluorescence Imaging

The PHEVD residues after the 7 day incubation and PHEVD film controls without incubation with PAO1 cells were labeled with acridine orange (Sigma-Aldrich, St. Louis, MO, USA) as previously described^72^ before the fluorescence imaging. Briefly, the washed PHEVD residues and controls were soaked in 10 mL of acridine orange solutions (0.5 mg mL^–1^ in DI water) with a pH of 3 (5 vol/vol % acetic acid) for 2 min at room temperature. Then, the stained PHEVD residues and controls were rinsed three times in DI water to remove excessive dyes before being imaged by a 40× lens on an Echo Revolve fluorescent microscope and a green fluorescence protein (GFP) filter set (excitation wavelength: 457–487 nm and emission wavelength: 502–538 nm).

### 5.7 RNA Sequencing

For those PAO1 cultures we harvested every 2 days, we spun down the PAO1 cells by centrifuging 1 mL of PAO1 cultures in 1.5 mL microcentrifuge tubes at 21,300 × *g* for 3 min. We transferred the supernatants into clean microcentrifuge tubes. Both supernatants and pellets were then quickly frozen using liquid nitrogen. The supernatants were stored at −80 °C before they were processed for ^1^H NMR and LC-MS. PAO1 cell pellets were shipped to Azenta (South Plainfield, NJ, USA) for RNA sequencing.

### 5.8 ^1^H NMR and LC-MS

Before the ^1^H NMR and LC-MS analyses, we extracted the chemicals by following metabolic extraction^73^ to minimize the chemical background. For metabolic extraction, we took frozen supernatants (harvested at days 2, 4, 6, and 7) from a −80 °C freezer and thawed them completely on ice (∼2–3 h). We prepared 50 vol/vol % methanol by mixing equal parts of methanol and DI water in 50 mL conical tubes. We mixed 0.25–0.5 mL of supernatants with 1–1.25 mL of 50% methanol. The mixtures were incubated at −80 °C for at least 2 h and then thawed again on ice. The mixtures were then centrifuged at 21,300 × *g* for 5 min at 4 °C. The supernatants were transferred to clean microcentrifuge tubes. Whenever pellet/debris remained, we resuspended them in 1 mL of DI water and centrifuged the mixtures again at 21,300 × *g* for 5 min at 4 °C. We pooled supernatants with the first extraction by mixing approximately 0.25 mL of each. We then added 1 mL of chloroform to the pooled supernatant and then briefly vortexed and centrifuged at 8,000 × *g* for 5 min to separate phases. The top aqueous phase was transferred to a new tube. For ^1^H NMR, we dried both the top and bottom aqueous phases under vacuum at room temperature overnight and stored them at −20 °C until use. The chemicals were dissolved in D_2_O before ^1^H NMR analysis. For LC-MS, both phases were stored directly at −20 °C until analysis. For the PHEVD films before and after PAO1 degradation (Figure 2a1 and c3–c6), they were dried under vacuum at room temperature and then dissolved in deuterated dimethyl sulfoxide (DMSO-d6) before being analyzed by ^1^H NMR. The ^1^H NMR (Bruker 400 MHz) and LC-MS (Agilent 6120B) characterizations were conducted at the Yale University West Campus Analytical Core (WCAC).

### 5.9 Size Exclusion Chromatography (SEC)

The molecular weight and polydispersity of synthetic polymers were determined by a size exclusion chromatography (SEC) system (TOSOH EcoSEC HLC-8320) equipped with a set of Phenomenex Phenogel 5μ, 1K–75K, 300 × 7.80 mm column in series with a Phenomenex Phenogel 5μ, 10K–1000K, 300 × 7.80 mm column following a guard column and two detectors including an RI detector and a UV detector. The measurements were performed using HPLC-grade dimethylformamide (DMF) containing 0.05 M LiBr as the eluent at a flow rate of 0.5 mL/min at 40 °C and a series of polystyrene standards for the calibration of the columns.

### 5.10 RT-qPCR

For the PAO1 cell pellets, we confirmed the RNA sequencing data via RT-qPCR. We first thawed the pellets taken from −80 °C from a freezer on ice. Then, we isolated RNA using a GeneJET RNA Purification Kit (Thermo Fisher Scientific, Waltham, MA, USA). We resuspended the thawed pellet thoroughly into 100 μL of freshly prepared TE buffer (10 mM Tris-HCl, pH 8.0; 1 mM EDTA) supplemented with lysozyme to a final concentration of 0.4 mg mL^–1^, vortexed it to dissolve, and incubated it for 5 min at 23 °C (pre-equilibrate the heat block) after a quick-spin. We then added 300 μL of lysis buffer freshly supplemented with 2 M DTT (1,4-dithio-dl-threitol), vortexed it for 15 s, and then added 180 μL of 96% ethanol and mixed it by slowly pipetting up and down 10 times. We loaded up to 700 μL of lysate onto the spin purification column and centrifuged it for 1 min at 12,000 × *g*. We discarded the flow-through and returned the column to the collection tube. We then washed the column with 700 μL of WB1 (with ethanol), followed with 600 and 250 μL of WB2 (with ethanol). Finally, we performed an additional empty spin at 12,000 × *g* for 5 min before air-drying the column at room temperature for ∼15 min. The column was place into a clean RNase-free 1.5 mL microcentrifuge tube before the RNAs were eluted by applying 100 μL of nuclease-free water to the center of the membrane, incubating for 5 min at room temperature, and centrifuging for 1 min at 12,000 × *g*. The RNA concentration and purity were evaluated by running RNA gels.

RNAs were cleaned by TURBO DNase (Thermo Fisher Scientific, Waltham, MA, USA) to remove DNAs right after the RNA isolation. Specifically, we first set aside 18 μL of the original RNA sample for gel comparison. To the remaining RNA, we added 0.1 volume of 10× TURBO DNase Buffer and 1 μL of TURBO DNase, and mixed it. For example, with ∼76 μL of RNA, we added ∼8 μL of buffer. The mixture was incubated at 37 °C for 30 min and then mixed with the DNase Inactivation Reagent by flicking or brief vortexing. Then, we added the inactivation reagent (use 0.1× volume or at least 2 μL, e.g., 8 μL for a 80 μL mixture), mixed it well, and incubated it for 5 min at 25 °C. We then flicked the tube 2–3 times to keep the reagent suspended. Finally, we centrifuged it at 10,000 × *g* for 1.5 min at 25 °C and carefully transferred the supernatant (RNA) to a fresh RNase-free tube without disturbing the pellet. The RNA concentration and purity were evaluated by running RNA gels.

The cleaned RNAs were quickly used to perform the reverse transcription using an iScript cDNA Synthesis Kit (Bio-Rad, Hercules, CA, USA). We first thawed the RNA on ice, flicked and quick-spun whenever needed, and reserved 9–18 μL of RNA for later gel verification. In a 96-well plate, we prepared each 20 μL reaction by adding nuclease-free H_2_O to each well first and then a variable volume of RNA (per ImageJ/Excel calculations), followed by 4 μL of 5× iScript Reaction Mix and 1 μL of iScript Reverse Transcriptase, keeping the enzyme addition last. We pipetted up and down three times to mix (tip at the bottom of the well to ensure complete delivery), sealed the plate, removed bubbles by gentle flicking and a brief spin, and ran the thermocycle as follows: 5 min hold at 26 °C, 46 °C for 20 min (reverse transcription), 95 °C for 1 min, and 4 °C hold, confirming that the total reaction volume of the instrument was set to 20 μL before starting. The cDNA concentration and purity were evaluated by running DNA gels. The cDNAs were stored at −80 °C until use.

For RT-qPCR, we kept an SYBR Green Master Mix kit (Thermo Fisher Scientific, Waltham, MA, USA) on ice (vortex thoroughly and quick-spin), thawed cDNA samples and primers on ice, vortexed, and quick-spun. We then prepared 20 μL reactions per well using Excel to calculate 10 ng of cDNA per well and added components in this order: nuclease-free H_2_O (variable to 20 μL), cDNA (per Excel), forward primer then reverse primer (typically 1.6 μL of each when using 10 μM working stocks to achieve a 800 nM reaction concentration; adjust to 0.6–1.6 μL of each for 300–800 nM as dictated by the primer datasheet), and finally 10 μL SYBR Green Master Mix (use 5 μL only if the instrument/protocol specifies a 10 μL total volume format). We set up at least duplicates (preferably triplicates) for each target gene. On the Thermo Fisher laptop, we loaded the ΔΔCt FAST template, updated master mix/primer targets, ensured that the reaction volume was set to 20 μL, saved the setup to the Thermo Fisher USB drive, powered on the QuantStudio, inserted the drive, and started the run. We analyzed data with the ΔΔCt method to quantify relative expression (fold up/downregulation) by comparing Ct values of each sample to the chosen calibrator after normalization to the housekeeping gene, *nadB*. The primers are listed in Table S6.

### 5.11 Enzyme Kinetics

We characterized the Chitinase kinetics by following a published protocol.^74^ Specifically, the activity of Chitinase (Chitinase from *Trichoderma viride*, 25 units; Sigma-Aldrich, St. Louis, MO, USA) was defined as the relative percentage reduction in the turbidity of the colloidal chitin (Biosynth International, Inc., Gardner, MA, USA) with versus without enzyme (control). The colloidal chitin solution (1% (w/v) Colloidal Chitin solution) was prepared by dissolving 10 mg in 1 mL of 50 mM Phosphate-Buffered Saline (PBS, pH 6.7). The relationship between the change in chitin concentration and turbidity absorbance at a wavelength of 510 nm was measured using a Synergy H1Multimode Reader (BioTek, Winooski, VT, USA; Figure S9). 20 μL of enzyme solution (1 unit in PBS with pH 7.4) was added to 1500 μL of 1% (w/v) colloidal chitin in 50 mM PBS (pH 6.7) and incubated at 28 °C for at least 5 h to repeat the previously published *V*_max_ (4.26 μg h^–1^) and K_M_ (2.92 mg mL^–1^)^74^ (Figure S10) and then at 37 °C for this study (Figure 6b). The absorbance of PBS, 1% (w/v) colloidal chitin only solution, and 1 unit enzyme only solution was tracked over time as control. One unit of Chitinase activity was defined as the amount of enzyme needed to reduce 5% turbidity of colloidal chitin.

PHEVD and PET films were converted into particles using a diamond sharpening stone (2000 grit). The diameters of the PHEVD and PET particles were measured using an Echo Revolve fluorescent microscope with 10× and 40× lens. The Chitinase kinetics of PHEVD and PET films were characterized by monitoring the change of their dry weight over time in PBS (pH 7.4) with and without 1 unit of Chitinase incubated at 37 °C. The Chitinase kinetics of PHEVD and PET particles were characterized by following the same procedure as that used for colloidal chitin as described above.

## Supplementary Material



## Notes

Comparison of thermal properties of PHEVD and representative petroleum-derived polymers; log2 fold changes of the genes that are involved in the hydrolytic degradation of amide bonds, the oxidative degradation of the imide ring, and the metabolic assimilation of degradation products; list of primers for RT-qPCR; synthetic scheme and ^1^H NMR spectrum of HEVD; synthetic scheme and ^1^H NMR spectrum of PHEVD; SEC trace of PHEVD; SEC trace of PHEVD; change of the dry weight of HDPE films after 7 days of incubation in LB with and without PAO1 cells; bright-field images of clean SDS solution and SDS solution with PHEVD residues after a 7 day biodegradation with PAO1, three washes, and 4 h of incubation in SDS solution at 37 °C with shaking at 200 rpm; particle distribution of PET and PHEVD particles; change of PET films over time with or without 1 unit of Chitinase in PBS with a pH of 7.4; change of turbidity of PET particles and Chitinase over time at 37 °C measured at an optical density of 510 nm; correlation between chitin concentration and absorbance at a wavelength of 510 nm; and change of turbidity of chitin and Chitinase over time at 28 °C measured at an optical density of 510 nm (PDF)
