# Supplementary material for: Design of a Chitinase-Responsive, Depolymerizable Petroleum-Derived Polymer for Circular and Antifouling Materials
Source: ACS Appl Polym Mater. 2026 Jun 3;8(12):9175–88. doi: 10.1021/acsapm.6c00740 (PMC13316867; doi:10.1021/acsapm.6c00740)
Supplement: Supplementary file 1 [file ap6c00740_si_001.pdf]

Supporting Information

Design of a Chitinase-Responsive, Depolymerizable Petroleum-Derived Polymer for Circular and Antifouling Materials

Cody J. Velikaneye[1,*], Sarah Kispert[1,*], Alexis Pishnyuk[1], Madison Kajuffa[1], Devin Fauver[1], Bisher Lpizra[1], Sneh Dagar[1], Safi Dapetel Balkissou Wouna[1], Kyle P. Buckley[1], Tarek Ibrahim[1], Hao Sun[1], Kristine Horvat[1], Chong Qiu[1], and Huan Gu[1,*]

[1]Department of Chemistry & Chemical Engineering and Biomedical Engineering, Tagliatela College of Engineering, University of New Haven, West Haven, CT 06516, United States

*Corresponding author

Huan Gu: Phone: 203-932-7000. Email: hgu@newhaven.edu

Table S1. Comparison of thermal properties of PHEVD and representative petroleum-derived polymers.

**Polymer**	**Glass transition temperature (T_g)**	**Thermal degradation onset***	**Reference**
**PHEVD**	Not detected	375–550 °C	This work
**Polyethylene (PE)**	Not detected	400–450 °C	[1, 2]
**Polyethylene terephthalate (PET)**	~70–80 °C	375-400 °C	[3, 4]

Table S2. Log2 fold changes of the genes that are involved in hydrolytic degradation of amide bonds.

Day 4				Day 6			
ID	log2-Fold Change	p-value	p-adj	ID	log2 Fold Change	p-value	p-adj
cbpD	3.17	4.4×10^{-11}	1.6×10^{-9}	*cbpD*	4.11	2.9×10^{-11}	8.4×10^{-10}
chiC	4.33	1.1×10^{-15}	7.7×10^{-14}	*chiC*	5.05	1.9×10^{-7}	2.4×10^{-6}
opdO	1.99	7.6×10^{-9}	1.8×10^{-7}	*opdO*	3.11	1.2×10^{-19}	1.3×10^{-17}
				opdH	1.39	1.8×10^{-6}	1.7×10^{-5}
aprA	1.73	2.8×10^{-5}	2.7×10^{-4}	*aprA*	2.84	2.7×10^{-8}	4.1×10^{-7}
aprD	1.95	2.5×10^{-8}	5.3×10^{-7}	*aprD*	2.18	7.2×10^{-7}	7.7×10^{-6}
aprE	2.14	1.9×10^{-10}	6.4×10^{-9}	*aprE*	2.15	2.6×10^{-7}	3.2×10^{-6}
aprF	1.75	1.9×10^{-6}	2.6×10^{-5}	*aprF*	1.66	1.1×10^{-4}	5.9×10^{-4}
aprI	2.61	8.4×10^{-17}	6.6×10^{-15}	*aprI*	2.54	1.4×10^{-6}	1.3×10^{-5}
				ampG	1.02	1.5×10^{-11}	4.6×10^{-10}
				fadD1	1.14	4.5×10^{-11}	1.2×10^{-9}
				faoA	2.25	7.8×10^{-11}	2.1×10^{-9}
				bdhA	1.23	6.6×10^{-4}	2.7×10^{-3}
				pckA	1.38	3.1×10^{-9}	5.9×10^{-8}
				pgm	1.75	6.2×10^{-31}	1.6×10^{-28}
glyA1	-3.54	1.7×10^{-42}	6.5×10^{-40}	*glyA1*	-1.39	2.0×10^{-4}	9.7×10^{-4}
				glyA2	1.47	2.0×10^{-3}	7.0×10^{-3}
				glyA3	1.93	4.4×10^{-17}	3.5×10^{-15}
				serA	1.40	2.0×10^{-9}	4.1×10^{-8}
				tauD	1.09	5.1×10^{-3}	1.5×10^{-2}

Table S3. Log2 fold changes of the genes that are involved in the oxidative degradation of the imide ring.

ID	log2-Fold Change	p-value	p-adj	ID	log2 Fold Change	p-value	p-adj
phzA1	3.08	9.1×10^{-9}	2.1×10^{-7}	phzA1	3.27	6.6×10^{-7}	7.2×10^{-6}
phzA2	2.59	1.2×10^{-11}	4.8×10^{-10}	phzA2	3.17	2.4×10^{-9}	4.9×10^{-8}
phzB1	3.93	7.8×10^{-6}	8.8×10^{-5}	phzB1	4.90	7.0×10^{-13}	2.8×10^{-11}
phzB2	3.61	2.5×10^{-11}	9.4×10^{-10}	phzB2	5.60	1.3×10^{-8}	2.2×10^{-7}
phzC1	2.62	9.2×10^{-13}	4.7×10^{-11}	phzC1	3.70	4.4×10^{-11}	1.2×10^{-9}
phzC2	2.52	2.0×10^{-12}	9.7×10^{-11}	phzC2	3.56	1.1×10^{-10}	2.7×10^{-9}
phzD1	3.55	1.3×10^{-10}	4.6×10^{-9}	phzD1	4.97	9.7×10^{-14}	4.9×10^{-12}
phzD2	3.49	1.0×10^{-9}	2.8×10^{-8}	phzD2	4.92	3.6×10^{-13}	1.6×10^{-11}
phzE1	3.16	3.6×10^{-10}	1.1×10^{-8}	phzE1	4.56	1.0×10^{-11}	3.2×10^{-10}
phzE2	3.13	1.4×10^{-9}	3.6×10^{-8}	phzE2	4.52	2.6×10^{-11}	7.7×10^{-10}
phzF1	3.05	4.6×10^{-9}	1.1×10^{-7}	phzF1	4.48	5.9×10^{-11}	1.6×10^{-9}
phzF2	3.10	4.0×10^{-10}	1.2×10^{-8}	phzF2	4.51	2.1×10^{-11}	6.3×10^{-10}
phzG1	2.64	5.8×10^{-8}	1.2×10^{-6}	phzG1	3.98	4.5×10^{-9}	8.4×10^{-8}
phzG2	2.47	2.3×10^{-9}	5.9×10^{-8}	phzG2	3.86	2.7×10^{-9}	5.2×10^{-8}
phzH	1.40	8.5×10^{-9}	2.0×10^{-7}	phzH	1.37	5.2×10^{-4}	2.2×10^{-3}
phzM	2.10	7.6×10^{-7}	1.1×10^{-5}	phzM	2.89	6.9×10^{-8}	9.6×10^{-7}
phzS	2.37	8.8×10^{-9}	2.0×10^{-7}	phzS	3.42	3.2×10^{-10}	7.6×10^{-9}
hcnA	2.84	8.8×10^{-9}	2.0×10^{-7}	hcnA	3.41	6.2×10^{-15}	3.8×10^{-13}
hcnB	3.14	6.6×10^{-9}	1.6×10^{-7}	hcnB	3.79	5.1×10^{-13}	2.2×10^{-11}
hcnC	3.01	2.5×10^{-8}	5.3×10^{-7}	hcnC	3.53	9.9×10^{-11}	2.6×10^{-9}
htpG	1.30	4.6×10^{-5}	4.1×10^{-4}	htpG	1.60	5.6×10^{-14}	3.0×10^{-12}
mmsA	1.79	7.3×10^{-13}	3.8×10^{-11}	mmsA	2.19	1.6×10^{-16}	1.15E-14
mmsB	1.59	8.0×10^{-5}	6.6×10^{-4}	mmsB	2.55	5.9×10^{-11}	1.59E-09
toxR	1.20	8.7×10^{-6}	9.7×10^{-5}	toxR	1.67	6.7×10^{-5}	3.90E-04
				trxB2	2.08	3.9×10^{-19}	4.0×10^{-17}
				katB	1.59	1.4×10^{-4}	7.1×10^{-4}
				gcdH	1.98	2.8×10^{-4}	1.3×10^{-3}
				glcE	1.16	2.2×10^{-4}	1.1×10^{-3}
				cobH	1.11	2.6×10^{-3}	8.9×10^{-3}
				hemH	1.38	2.7×10^{-35}	1.4×10^{-32}
				mqoB	1.30	7.5×10^{-26}	1.4×10^{-23}
				aceA	1.53	2.4×10^{-17}	2.0×10^{-15}
				phoP	1.35	1.2×10^{-7}	1.6×10^{-6}
				phoQ	1.18	4.5×10^{-6}	3.9×10^{-5}

Table S4. Log2 fold changes of the genes that are involved in the metabolic assimilation of degradation products.

Day 4				Day 6			
ID	log2-Fold Change	p-value	p-adj	ID	log2 Fold Change	p-value	p-adj
acsA	1.18	1.0×10^{-2}	3.5×10^{-2}				
ampDh3	1.18	1.0×10^{-2}	4.2×10^{-2}				
moaB1	1.09	7.0×10^{-4}	4.2×10^{-3}				
nosL	1.13	5×10^{-3}	2.1×10^{-2}				
aruF	1.14	1.0×10^{-4}	9.9×10^{-4}				
ambB	2.87	1.4×10^{-9}	3.6×10^{-8}	*ambB*	3.39	5.2×10^{-9}	9.5×10^{-8}
ambC	3.18	9.3×10^{-11}	3.2×10^{-9}	*ambC*	3.87	7.6×10^{-10}	1.7×10^{-8}
ambD	3.34	4.8×10^{-10}	1.5×10^{-8}	*ambD*	4.08	2.9×10^{-5}	1.9×10^{-4}
ambE	2.88	8.8×10^{-11}	3.1×10^{-9}	*ambE*	3.80	1.8×10^{-8}	2.9×10^{-7}
atuA	1.36	1.8×10^{-6}	2.5×10^{-5}	*atuA*	1.55	1.3×10^{-18}	1.2×10^{-16}
atuB	1.13	7.0×10^{-4}	4.4×10^{-3}	*atuB*	1.49	2.5×10^{-13}	1.0×10^{-11}
fabH2	2.50	2.2×10^{-11}	8.4×10^{-10}	*fabH2*	3.48	1.5×10^{-13}	7.1×10^{-12}
cysA	1.30	2.0×10^{-10}	6.6×10^{-9}	*cysA*	1.78	8.6×10^{-11}	2.3×10^{-9}
				cysT	1.25	1.2×10^{-4}	6.3×10^{-4}
cysW	1.03	1.3×10^{-5}	1.4×10^{-4}	*cysW*	1.34	2.2×10^{-7}	2.7×10^{-6}
gcvH2	1.08	1.0×10^{-3}	7.7×10^{-3}	*gcvH2*	2.84	1.2×10^{-5}	8.9×10^{-5}
gcvP2	1.23	5.0×10^{-4}	3.3×10^{-3}	*gcvP2*	3.23	1.4×10^{-6}	1.3×10^{-5}
hpcG	1.80	5.9×10^{-8}	1.2×10^{-6}	*hpcG*	1.76	7.3×10^{-7}	7.8×10^{-6}
				cynT	1.65	6.0×10^{-12}	1.9×10^{-10}
				cysT	1.25	1.2×10^{-4}	6.3×10^{-4}
sbp	1.24	6.2×10^{-5}	5.3×10^{-4}	*sbp*	1.98	2.80E-04	1.32E-03
pheC	1.16	4.9×10^{-10}	1.5×10^{-8}	*pheC*	1.51	5.3×10^{-9}	9.5×10^{-8}
wzx	1.22	3.7×10^{-6}	4.7×10^{-5}	*wzx*	1.29	5.5×10^{-6}	4.6×10^{-5}
rimJ	1.12	5.0×10^{-3}	1.9×10^{-2}	*rimJ*	1.31	3.23E-04	1.49E-03
				phnW	1.46	5.6×10^{-14}	3.0×10^{-12}
				purB	1.82	9.1×10^{-10}	2.0×10^{-8}
				purH	1.42	3.1×10^{-6}	2.7×10^{-5}
				pyrF	1.12	6.0×10^{-7}	6.7×10^{-6}
				pyrH	1.18	1.6×10^{-6}	1.5×10^{-5}

Table S5. Log2 fold changes of the genes that are involved in the metabolic assimilation of degradation products.

Day 4				Day 6			
ID	log2-Fold Change	p-value	p-adj	ID	log2 Fold Change	p-value	p-adj
cupC2	1.20	6.7×10^{-6}	7.7×10^{-5}				
flp	1.53	1.0×10^{-3}	6.3×10^{-3}	*flp*	1.94	6.6×10^{-3}	1.9×10^{-2}
tadA	1.16	9.6×10^{-7}	1.4×10^{-5}	*tadA*	1.49	3.4×10^{-5}	2.2×10^{-4}
				tadZ	1.05	7.1×10^{-5}	4.1×10^{-4}
oprH	1.02	2.0×10^{-3}	1.1×10^{-2}	*oprH*	1.46	4.8×10^{-9}	8.8×10^{-8}
oprJ	2.77	5.9×10^{-26}	1.2×10^{-23}	*oprJ*	2.57	6.3×10^{-35}	3.0×10^{-32}
pslA	1.11	8.0×10^{-3}	2.9×10^{-2}	*pslA*	1.05	1.6×10^{-2}	4.1×10^{-2}
pslB	1.94	1.7×10^{-6}	2.5×10^{-5}	*pslB*	2.31	6.3×10^{-9}	1.1×10^{-7}
pslC	1.82	1.5×10^{-6}	2.2×10^{-5}	*pslC*	2.16	1.3×10^{-8}	2.1×10^{-7}
pslD	1.86	3.2×10^{-7}	5.3×10^{-6}	*pslD*	2.34	4.7×10^{-10}	1.1×10^{-8}
pslF	1.32	1.9×10^{-6}	2.7×10^{-5}	*pslF*	1.62	5.4×10^{-7}	6.0×10^{-6}
pslG	1.28	6.5×10^{-18}	6.2×10^{-16}	*pslG*	1.27	3.2×10^{-8}	4.8×10^{-7}
pslH	1.26	3.8×10^{-10}	1.2×10^{-8}	*pslH*	1.21	1.7×10^{-6}	1.6×10^{-5}
pslI	1.07	6.0×10^{-9}	1.5×10^{-7}	*pslI*	1.29	8.3×10^{-7}	8.7×10^{-6}
pslJ	1.34	2.1×10^{-12}	1.0×10^{-10}	*pslJ*	1.36	1.2×10^{-6}	1.2×10^{-5}
				pslK	1.07	6.6×10^{-6}	5.4×10^{-5}
lecA	2.04	2.0×10^{-4}	1.5×10^{-3}	*lecA*	2.36	1.0×10^{-4}	5.5×10^{-4}
lecB	3.41	5.2×10^{-5}	4.6×10^{-4}	*lecB*	3.42	5.3×10^{-7}	5.9×10^{-6}
lasA	2.84	2.2×10^{-8}	4.8×10^{-7}	*lasA*	3.88	6.6×10^{-13}	2.7×10^{-11}
lasB	3.62	5.6×10^{-5}	4.9×10^{-4}	*lasB*	5.20	9.9×10^{-14}	4.9×10^{-12}
pqsA	1.26	6.0×10^{-3}	2.5×10^{-2}	*pqsA*	2.02	1.5×10^{-5}	1.1×10^{-4}
pqsB	1.10	1.0×10^{-2}	4.1×10^{-2}	*pqsB*	1.90	4.7×10^{-5}	2.93E-04
				pqsC	1.78	2.1×10^{-4}	1.0×10^{-3}
				pqsD	1.62	5.8×10^{-4}	2.4×10^{-3}
				pqsE	1.26	7.73E-03	2.17E-02
pqsH	1.46	7.9×10^{-8}	1.5×10^{-6}				
				pelB	1.12	7.0×10^{-6}	5.7×10^{-5}
qscR	1.05	4.0×10^{-9}	9.8×10^{-8}	*qscR*	1.15	1.2×10^{-3}	4.6×10^{-3}
qteE	1.85	5.7×10^{-7}	8.8×10^{-6}	*qteE*	1.83	6.8×10^{-6}	5.6×10^{-5}
psrA	1.44	9.0×10^{-4}	5.2×10^{-3}	*psrA*	2.29	6.0×10^{-12}	2.0×10^{-10}
				vgrG1	1.18	2.0×10^{-7}	2.6×10^{-6}
fliC	1.20	2.0×10^{-3}	1.0×10^{-2}				
bdlA	1.52	1.4×10^{-7}	2.5×10^{-6}	*bdlA*	1.11	1.3×10^{-4}	7.0×10^{-4}

Table S6. List of primers for RT-qPCR.

Gene	Forward primer (5’-3’)	Reverse primer (5’-3’)
nadB	**ATGTCGATGCCGAAGTCCAG**	**GGATCGACTGCGTCTACCTG**
chiC	**GCTGACGATGAAGTGCTTGC**	**CGTCTGGTGGAAACCTACGG**

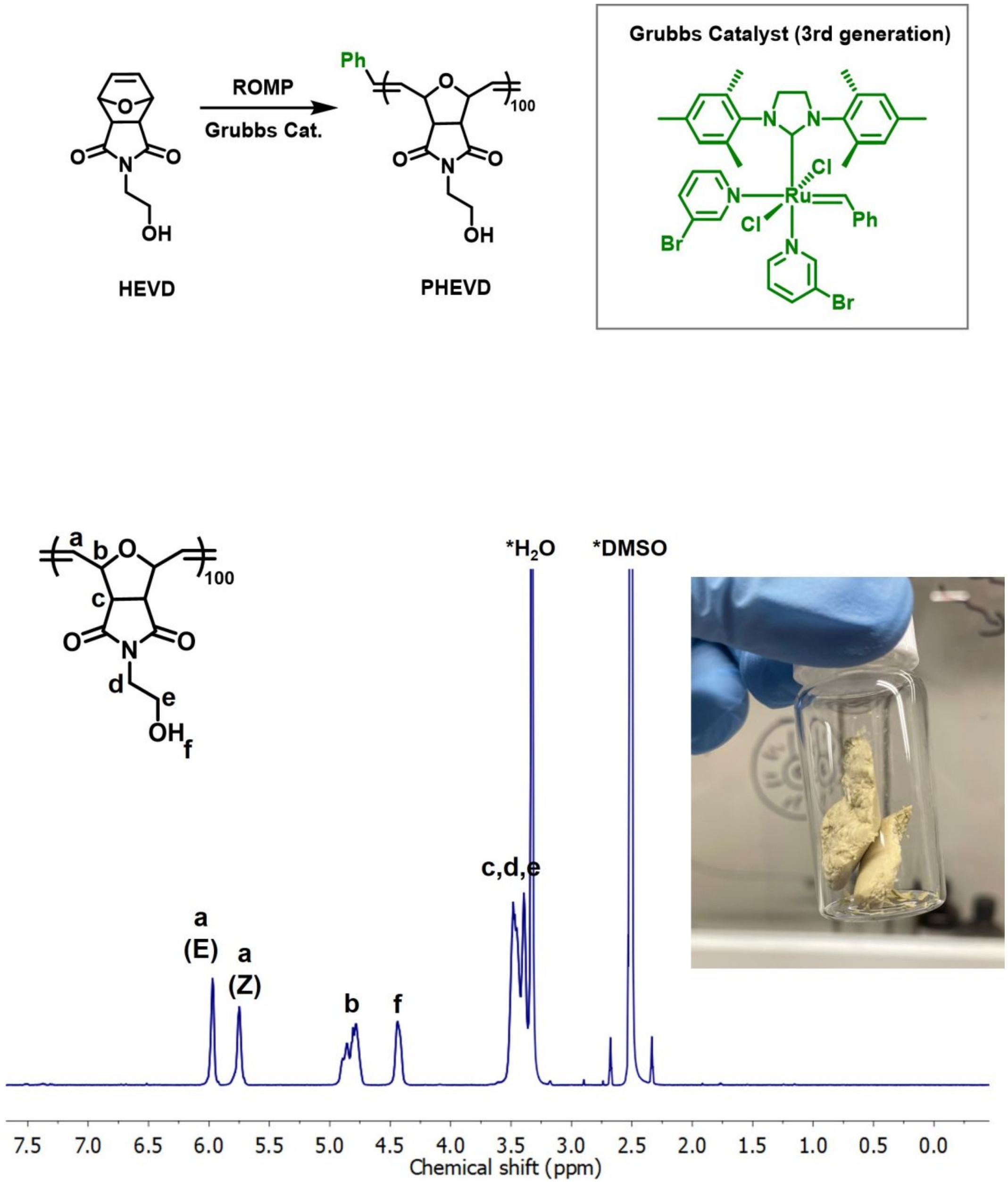

Figure S1. Synthetic scheme and ^{1}H-NMR spectrum of PHEVD. A photo of the polymer product is shown in the inset of the figure.

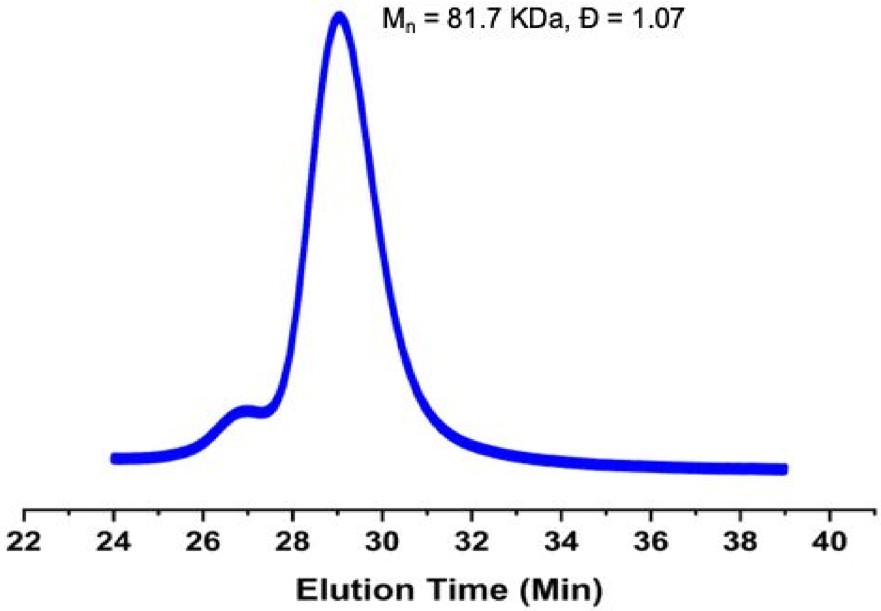

Figure S2. SEC trace of PHEVD.

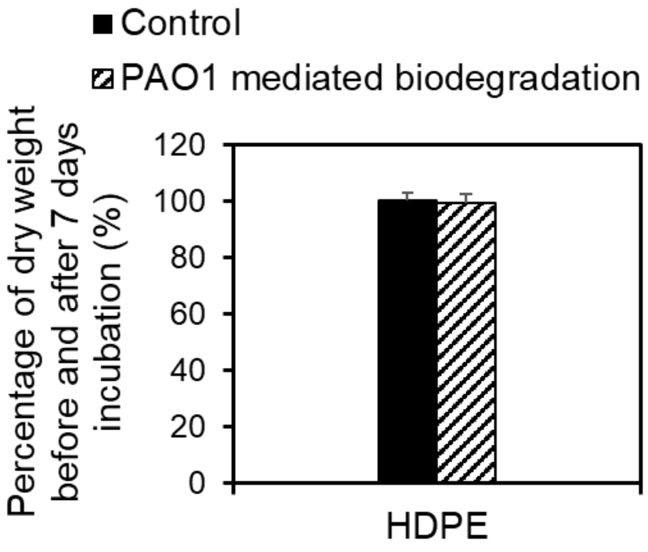

Figure S3. Change of HDPE films' dry weight after 7 days incubation in LB broth with and without PAO1 cells.

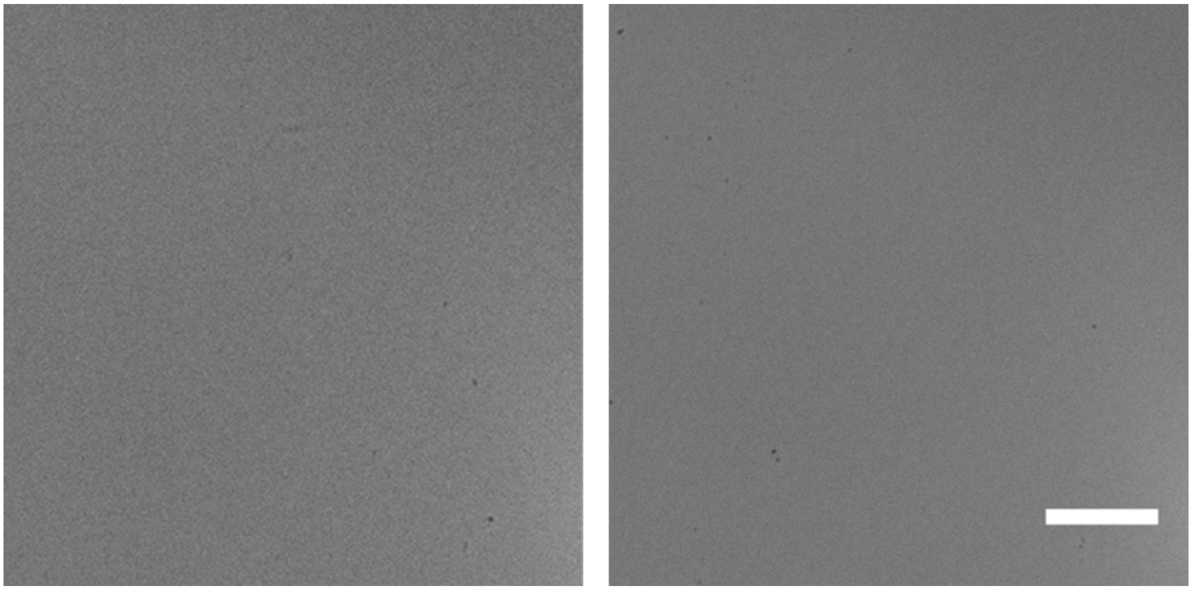

Figure S4. Brightfield images of clean SDS solution (Left) and SDS solution with PHEVD residues after a 7-day biodegradation with PAO1 solutions, three washes in deionized water (DI water), and 4 h incubation in SDS solution at 37 °C with shaking at 200 rpm (Right). Bar = 20 μm. If microplastics were present, significantly more black dots would be expected in the brightfield image on the right.

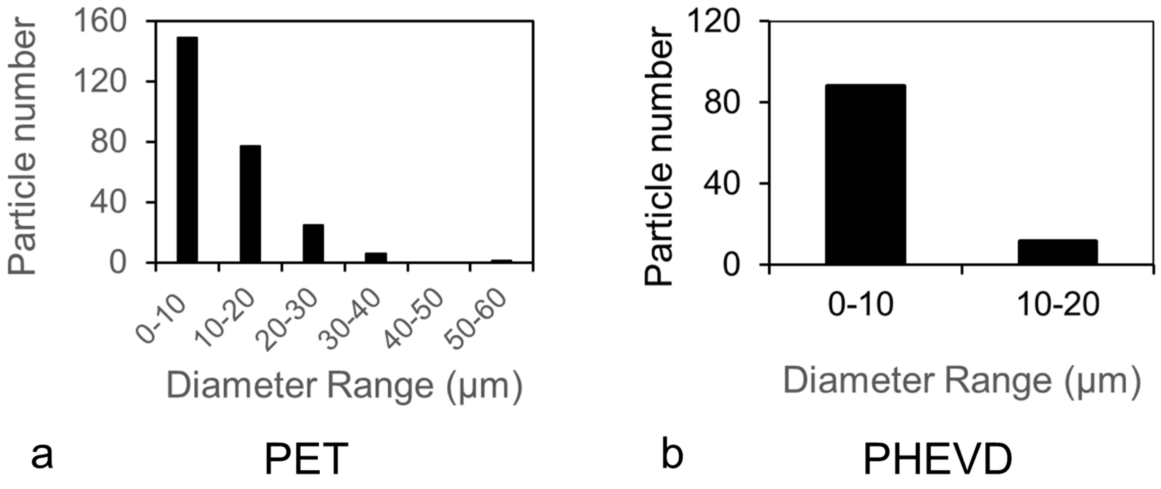

Figure S5. Particle distribution of PET (a) and PHEVD (b) particles. For PET particles, the diameter of 258 particles was measured. For PHEVD particles, the diameter of 93 particles were measured.

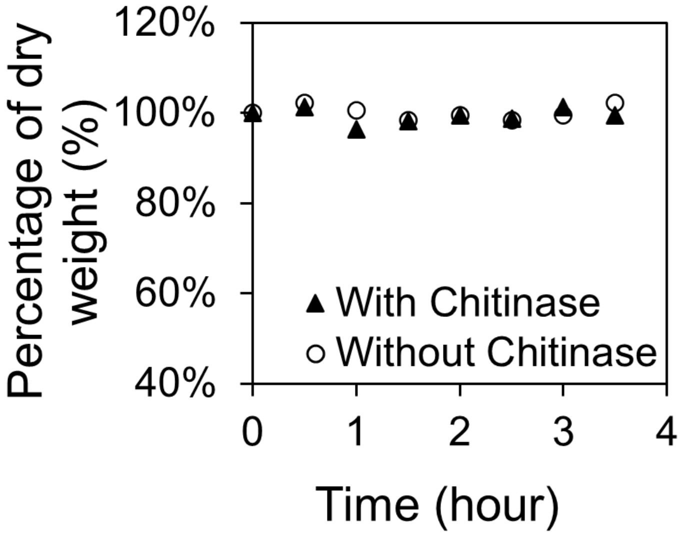

Figure S6. The change of PET films over time with or without 1 unit of chitinase in PBS with a pH of 7.4.

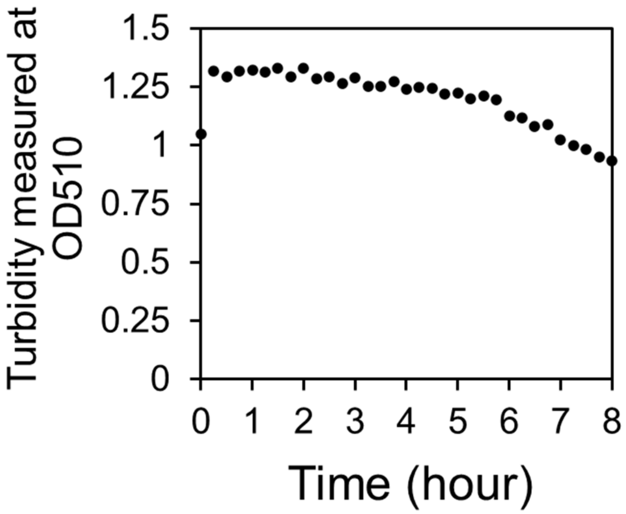

Figure S7. The change of turbidity of PET particles and chitinase overtime at 37 °C measured at the optical density of 510 nm.

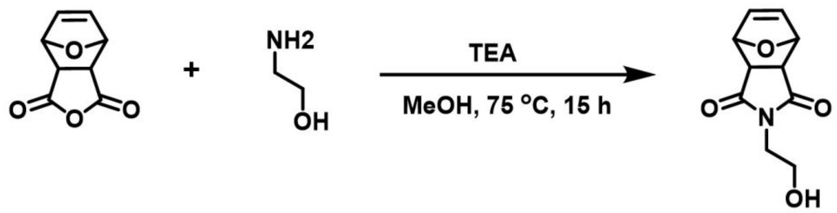

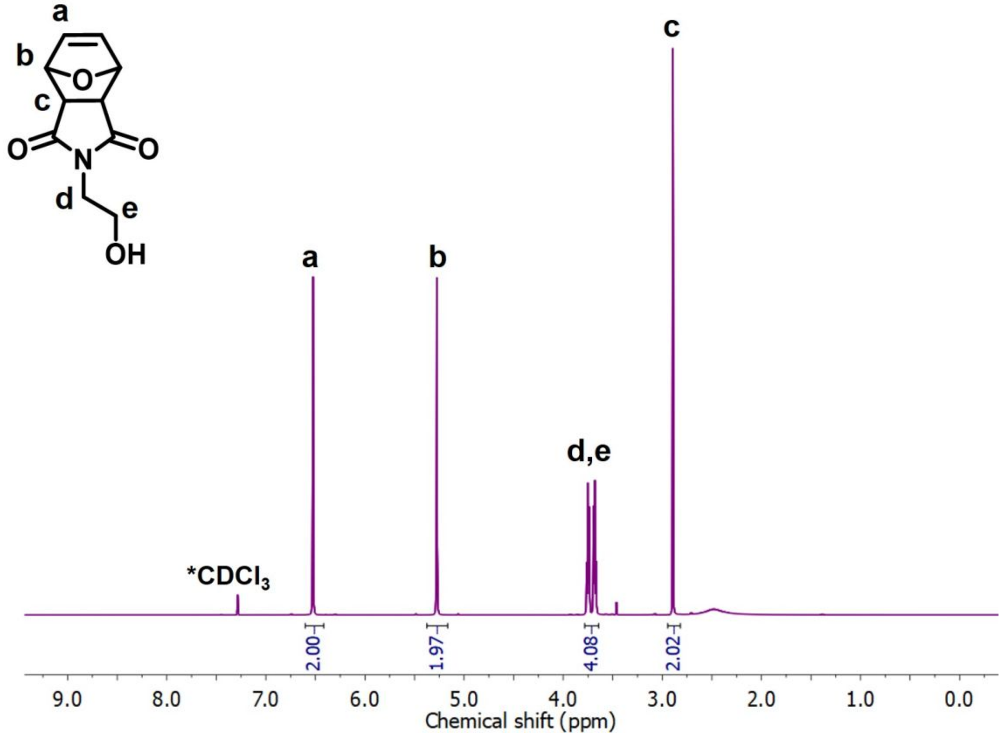

Figure S8. Synthetic scheme and ^{1}H-NMR spectrum of HEVD.

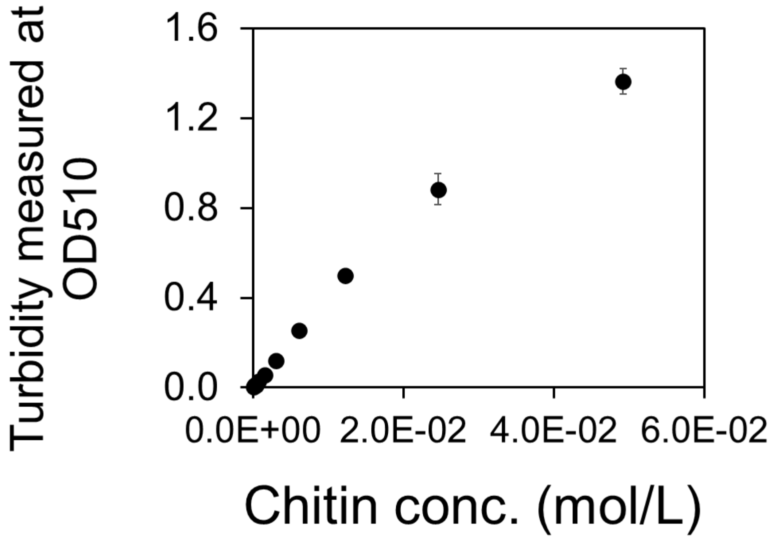

Figure S9. The correlation between chitin concentration and absorbance at the wavelength of 510 nm.

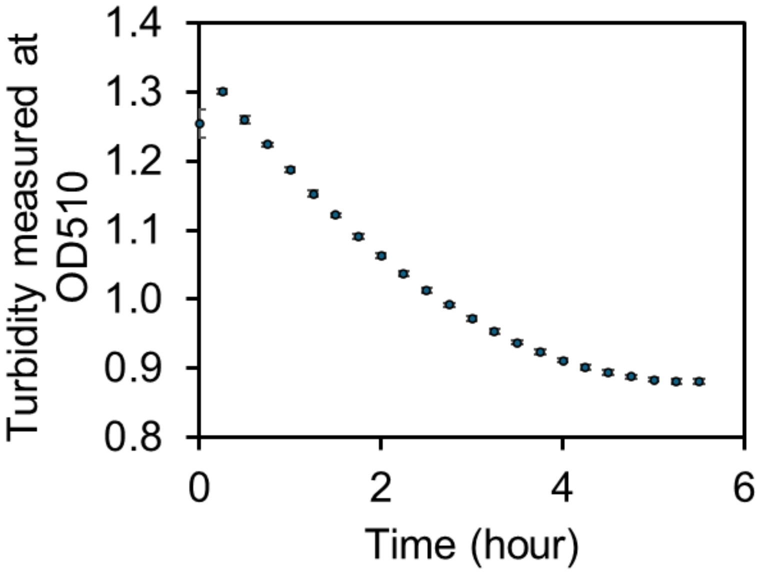

Figure S10. The change of turbidity of chitin and chitinase overtime at 28 °C measured at the optical density of 510 nm. (N = 3)

References:

(1) Miskolczi, N., Bartha, L. and Deák, Gy. Thermal degradation of polyethylene and polystyrene from the packaging industry over different catalysts into fuel-like feed stocks. *Polymer Degradation and Stability* **2006**, *91* (3), 517-526.

(2) Murata, K. Thermal Degradation of High Density Polyethylene. *Transactions of K. Murata Research Lab* **2020**, *7*.

(3) Botelho, G., Queiros, A., Literal, S., Gijsman, P. Studies on thermal and thermo-oxidative degradation of poly(ethylene terephthalate) and poly(butylene terephthalate). *Polymer Degradation and Stability* **2001**, *74* (1), 39-48.

(4) Chiu, S.J., and Cheng, W. H. Thermal degradation and catalytic cracking of poly(ethylene terephthalate). *Polymer Degradation and Stability* **1999**, *63* (3), 407-412.
